# Exploring the bidirectional relationship between depressive disorder and dyslipidemia: a systematic review and meta-analysis

**DOI:** 10.3389/fpsyt.2025.1498773

**Published:** 2025-12-10

**Authors:** Xiaxia Jin, Chaobin Kang, Yanrong Lu, Yifan Yang, Feng Zhou, Tao Gao, Xiaochun Liu, Yongmei Yan

**Affiliations:** 1 National Resource Center for Chinese Materia Medica, China Academy of Chinese Medical Sciences, Beijing, China; 2 Department of Acupuncture, Shaanxi Provincial Hospital of Traditional Chinese Medicine, Xi’an, China; 3 Guanganmen Hospital Affiliated to China Academy of Chinese Medical Sciences, Beijing, China; 4 The Affiliated Hospital of Shaanxi University of Chinese Medicine, Xianyang, China; 5 College of Acupuncture and Massage, Shaanxi University of Chinese Medicine, Xianyang, China; 6 Department of Rehabilitation, Xi'an TCM Hospital of Encephalopathy, Xi’an, China

**Keywords:** depressive disorder, bidirectional relationship, serum 5-HT, dyslipidemia, a systematic, meta-analysis

## Abstract

**Objective:**

To explore the bidirectional association between dyslipidemia and depression, and the potential predictive role of lipid level changes for depression onset.

**Methods:**

A systematic search of the Cochrane Library, Web of Science, Embase, and PubMed databases was conducted to identify cohort studies on blood lipids and 5-hydroxytryptamine (5-HT) parameters related to depression, from database inception to May 2024. Data were analyzed using Stata 14.0 software.

**Results:**

Seven studies were included in the analysis. Serum high-density lipoprotein (HDL) and triglyceride levels are significantly associated with an increased risk of depression (OR = 1.28, 95% CI: 1.07 - 1.53; OR = 1.41, 95% CI: 1.20 - 1.66, *P* < 0.05). However, depressive symptoms do significantly affect serum HDL, LDL, triglyceride, or total cholesterol levels (ORs = 0.88, 1.05, 1.05, 1.11; 95% CIs: 0.58 - 1.35, 0.88 - 1.24, 0.91 - 1.21, 0.9 - 1.32, *P* > 0.05).

**Conclusion:**

Based on the present findings, changes in serum HDL and triglyceride levels are significantly linked to depression incidence. Monitoring these two lipid parameters may aid in early identification of at-risk individuals and enhance the prognosis and quality of life for depressed patients through timely interventions.

**Systematic Review Registration:**

https://www.crd.york.ac.uk/prospero/, identifier CRD42024542833.

## 1 Introduction

As a major global public health challenge, depression contributes substantially to the global disease burden (1)— its core symptoms, defined by the Diagnostic and Statistical Manual of Mental Disorders (DSM-5), directly impair individual functioning and societal productivity. These key symptoms include persistent low mood, anhedonia (loss of interest or pleasure in previously enjoyable activities), generalized fatigue, psychomotor retardation or cognitive slowing, difficulty concentrating, and memory decline; In some cases, individuals may experience hallucinations, delusions, or even suicidal tendencies (2, 3). Together, these symptoms often lead to disrupted daily life, reduced work efficiency, and increased demand for healthcare services.

Fundamentally, depression is a multifactorial disease, and its pathogenesis involves three interrelated dimensions: biology (genetics, neurotransmitter imbalances) (4), psychology (chronic stress, negative cognitive patterns) (5), and society (interpersonal problems, socioeconomic status) (6). Despite extensive research, there remains no unified consensus in the academic community on the exact pathogenesis of depression, as its development typically arises from the complex interplay of the above factors rather than a single cause. In recent years, with advances in pathophysiological research, immune-inflammatory responses and metabolic abnormalities have gradually been confirmed as potential core mechanisms underlying the development of depressive disorders (7). Among these, abnormal serotonin (5-HT) levels and dyslipidemia serve as two key biological indicators. The association and interaction of these indicators with depression have become crucial scientific questions that urgently need clarification in the field.

Serotonin is an important neurotransmitter that plays a critical role in numerous physiological processes, including platelet aggregation, pain, sleep, appetite, muscle contraction, mood, and compulsive behaviors (8). Targeting the serotonin system is a key strategy for developing new potential antidepressants. Serotonin and its receptors are distributed in the central nervous system (CNS), peripheral nervous system (PNS), and multiple non-neuronal tissues, such as those in the gut, cardiovascular system, and blood. Serotonin can bind to cell-surface 5-HT1B receptors to regulate various physiological functions (e.g., pain, sleep, mood, and memory), and its levels have been associated with anxiety, depression, and schizophrenia (9). It has been reported that 5-HT2A receptor density is significantly increased in the cortex of postmortem brain tissue from depressed patients and individuals who died by suicide. This increase may be a compensatory response by the brain to reduced serotonin levels in depressed patients, achieved by upregulating 5-HT2A receptor density (10). Long-term administration of some antidepressant drugs downregulates 5-HT2A receptor density and exerts antidepressant effects by modulating subcortical circuits in the forebrain (11). These findings support a link between 5-HT2A receptor regulation and the therapeutic mechanism of these antidepressants; however, evidence for a direct association with depression pathogenesis remains to be supplemented. Vargas MV et al. (12)demonstrated that primary localization of 5-HT2ARs in cortical neurons is intracellular, cellular import of serotonin leads to structural plasticity and antidepressant-like effects.

The traditional monoamine hypothesis posits a link between brain monoamine neurotransmitter levels and depression onset, which laid the foundation for early antidepressant development but is now considered overly simplified—for instance, it cannot explain the delayed efficacy of monoamine-targeting drugs or why many patients fail to respond. Recent studies have updated this view by situating the monoamine system within a multi-mechanism network, with neuroinflammation as a key regulatory node. In depressed patients, elevated pro-inflammatory cytokines (e.g., IL-6, TNF-α) disrupt monoamine homeostasis: they inhibit tryptophan hydroxylase (reducing 5-HT synthesis) and activate indoleamine 2,3-dioxygenase (shifting tryptophan metabolism to neurotoxic metabolites), exacerbating monoamine imbalance (13, 14). Conversely, monoamines (especially 5-HT) modulate glial activation to regulate neuroinflammation, forming a bidirectional loop (15, 16). Notably, 5-HT2A receptors, which regulate monoamine release, may further mediate this ‘inflammation-monoamine’ crosstalk (17), linking their function to both depression pathogenesis and antidepressant mechanisms more precisely.

In the central nervous system, lipids are critical for maintaining the structure, function, and membrane integrity of various cells. Specifically, cholesterol—a key lipid component—modulates cellular signaling pathways and has been implicated in both the mechanism of antidepressant action and the regulation of emotional stability. Since blood cholesterol levels correlate with brain cholesterol levels, a decrease in neuronal membrane lipids can reduce serotonin (5-HT) receptor levels in the membrane, and this reduction is associated with increased risks of depression and suicide (18). This lipid-5-HT axis may further interact with 5-HT-related receptor complexes, which are mechanisms closely linked to major depressive disorder. For instance, Borroto-Escuela DO et al. (19) demonstrated that serotonin heteroreceptor complexes, which mediate signal integration in neurons and astrocytes, are highly relevant to the pathogenesis of major depressive disorder. Moreover, Ambrogini P et al. (20) found that 5-HT1A receptor-FGFR1 heteroreceptor complexes differentially modulate G protein-coupled inwardly rectifying potassium (GIRK) currents in the dorsal hippocampus and dorsal raphe nucleus serotonin neurons between control rats and a genetic depression model. These findings suggest that the function of 5-HT receptor complexes may be altered in the context of depression-related lipid/5-HT dysregulation. Beyond these molecular mechanisms, neuroplasticity changes in key brain regions also contribute to depression pathogenesis, with the hippocampus, prefrontal cortex, and amygdala being core areas involved (21). Notably, these neuroplasticity changes are regulated by multiple systems, including glutamatergic signaling, neurotrophic factors, monoamine neurotransmitters (e.g., 5-HT), and neuroinflammation—hinting at potential crosstalk between lipid/5-HT pathways and neuroplasticity regulation in depression.

In addition to the neurotransmitter system, lipid disorders in metabolic abnormalities have also been shown to be closely associated with depression. Studies have demonstrated that lipid metabolism disorders—specifically, lower levels of total cholesterol and LDL-C—are linked to an increased risk of suicide in patients with depression, making these lipid abnormalities among the high-risk factors for suicide in this population (22, 23). These reduced lipid levels may serve as potential biomarkers for predicting suicide attempts in patients with depression. The study by Nikolić V et al. helps elucidate a potential biological mechanism connecting dietary fiber intake to lipid profiles, thereby providing mechanistic support for the observed association between low HDL-cholesterol levels and depression (24). Recent advances in biomarker research have also provided new insights into the diagnosis and management of depression. These advances not only emphasize electrophysiological markers of depression (25) and genetic and epigenetic factors that underlie neurobiological and inflammatory pathways (26), but also suggest potential crosstalk between lipid metabolism and these neurobiological and inflammatory pathways in the pathogenesis of depression.

In specific populations, this association exhibits significant gender and age differences—for example, Yang et al. (27) found that women are more likely than men to develop dyslipidemia following antidepressant therapy, characterized by elevated triglyceride and LDL-C levels, with these elevations being more pronounced in women. Interestingly, high HDL-cholesterol levels in middle-aged adults have been associated with an increased risk of depression, and a relationship has been observed between depression and levels of total cholesterol and LDL-C (28). Furthermore, Mehdi SMA et al. confirmed, based on multi-generational depression cohorts and national health and nutrition survey data, that depression is significantly associated with lipid disorders (29). This provides empirical evidence at the population level for the involvement of lipid metabolism—particularly abnormal HDL-cholesterol levels—in the pathological process of depression.

At the mechanistic level, short-term population-based follow-up studies have shown that metabolic and hunger-related hormones (e.g., growth hormone, cortisol, leptin, ghrelin) directly affect serum lipid levels (30),or interact synergistically with immune inflammatory responses to jointly promote the occurrence and aggravation of depressive symptoms. Additionally, the use of antidepressant drugs has also been reported to alter lipid metabolism (31), which further intensifies the interaction between metabolic and hunger-related hormones and immune inflammatory responses. It is worth noting that metabolic syndrome is a pathological state characterized by multiple risk factors, including obesity, hypertension, dyslipidemia, and abnormal glucose metabolism. It promotes the development of cardiovascular and cerebrovascular diseases, as well as diabetes mellitus, and importantly, its association with depression has also been linked to lipid metabolism abnormalities (32–34). Studies have indicated that the association between depression and dyslipidemia is similar in strength and direction across different ages (35). Hence, lipid levels have the potential to be biomarkers for the diagnosis, classification, and prognosis of depression. However, there is still no definitive conclusion about the relationship between depression and dyslipidemia at different ages, and more studies are needed to explore this relationship.

Collectively, a large number of studies have confirmed correlations between lipid levels, 5-HT levels, and depression; however, the association between specific lipid parameters (e.g., HDL, LDL, triglycerides) and 5-HT-related indices and depression risk has rarely been investigated, and few studies have analyzed the bidirectional and causal relationships among these factors. Therefore, this study explored the bidirectional and causal relationships between depression and both serum 5-HT levels and dyslipidemia via a meta-analysis, thereby providing reliable evidence for the prevention and treatment of depression and a theoretical basis for further investigating its pathogenesis.

## 2 Research data and methods

### 2.1 Study inclusion and exclusion criteria

Inclusion criteria (1): Studies that measure depression using standardized self-report scales or diagnostic assessments. The scales include the 4-item version of the General Health Questionnaire (GHQ) (36, 37), (score ≥ 4 indicates depressive symptoms), the Center for Epidemiologic Studies Depression Scale (CES-D) (38), (a valid tool with a threshold score of 16 indicating clinically significant depressive symptoms), the 17-item Hamilton Depression Rating Scale (HDRS) (39), (score ≥ 18 reflects moderate-to-severe depression), and the Mini International Neuropsychiatric Interview (MINI) (40), (a structured diagnostic interview for psychiatric disorders, including depression). For diagnostic assessments, studies must use ICD-10 codes or DSM-IV criteria to confirm diagnoses (41) (2). Studies that include exposure factors as serum levels of 5-hydroxytryptamine (5-HT) and lipid parameters (total cholesterol, triglycerides, high-density lipoprotein cholesterol [HDL], low-density lipoprotein cholesterol [LDL], very-low-density lipoprotein cholesterol [VLDL]), with at least one parameter reported per study. Venous blood is collected from participants after an ≥ 12-hour fast. Serum levels of total cholesterol, HDL, triglycerides, and 5-HT are determined via conventional enzymatic methods. Studies must clearly report the units of each parameter (e.g., total cholesterol: mmol/L; serum 5-HT: ng/mL) to ensure accurate data synthesis in the meta-analysis and avoid unit inconsistencies (3). Studies with a longitudinal cohort design (4). Manuscripts published in English.

Exclusion Criteria (1): Studies including subjects with major chronic diseases (e.g., rheumatism, cancer), severe unstable medical conditions (e.g., advanced organ failure), diagnosed mental disorders other than unipolar depression (e.g., bipolar disorder, schizophrenia), or specific populations (e.g., postpartum women), except for studies including patients diagnosed with unipolar depression (2). Studies involving non-human subjects (3). Manuscripts without reported outcome data, and for which authors cannot provide such data (4). Data reported in meetings, abstracts, editorials, or letters (not full-text research articles).

### 2.2 Study retrieval strategies

We searched the Cochrane Library, Web of Science, Embase, and PubMed databases. Specifically, we searched for cohort studies investigating the association between serum serotonin (5-HT), total cholesterol, triglycerides, high-density lipoprotein cholesterol (HDL), low-density lipoprotein cholesterol (LDL), and very-low-density lipoprotein cholesterol (VLDL) and depression. The search was restricted to English-language publications (including full-text articles and conference proceedings).

The search strategy was developed in accordance with the guidelines of The Cochrane Handbook for Systematic Reviews of Interventions. English search terms included “depression”, “triglycerides”, “cholesterol”, “lipids”, “high-density lipoprotein cholesterol”, “low-density lipoprotein cholesterol”, and “serotonin” (the detailed retrieval process was provided in the supplementary material). A combination of MeSH terms (Medical Subject Headings) and free-text terms was used for the search. This study was registered in PROSPERO (International Prospective Register of Systematic Reviews) with the registration number CRD42024542833.

### 2.3 Study screening and data extraction

Two researchers independently read the articles according to the inclusion and exclusion criteria and then screened them to exclude articles with small sample sizes (n < 50), duplicate reports, or no original data. The information to be extracted included the following (1): Basic information: article title, author(s), publication year, sample size, study population, and population source (2); Study subject details: age, gender, and other demographic characteristics (3); Outcome data: number of participants in the exposed and non-exposed groups, original values of the exposure factors (serum 5-HT and lipid parameters), and reported Odds Ratio (OR) [Relative Risk (RR) was pre-specified as a potential effect size metric for extraction, given its common use in cohort studies. However, no included studies reported RR; we therefore synthesized and presented ORs to ensure accuracy and avoid metric conversion bias] or 95% Confidence Intervals (CI). The two researchers cross-checked the extracted content and entered the extracted data into a standardized database.

### 2.4 Evaluation of article quality

We used the Newcastle-Ottawa Scale (NOS) to assess the methodological quality of the included studies. The NOS consists of 8 items across 3 dimensions, each focusing on a key aspect of study quality (1): participant selection (4 items) (2); between-group comparability (1 item, maximum score of 2) (3); outcome measure assessment (3 items, 1 point each). The total possible score ranges from 0 to 9. A total score of ≥ 7 was defined as high methodological quality, 4–6 as moderate quality, and ≤ 3 as low quality; the higher the score, the better the methodological quality of the included studies.

### 2.5 Statistical analysis

Meta-analysis of the associations between serum lipid/(5-HT) levels and depression was performed using Stata 14.0 software. Count data were analyzed using RR or OR as effect sizes, and their 95% CI were reported. “Count data” specifically refers to dichotomous outcome variables quantified by event counts in the included longitudinal studies. These primarily include two core outcomes (1): incident depressive disorder (i.e., counting participants who developed depression during follow-up versus those who did not) and (2) incident dyslipidemia (counting participants who developed dyslipidemia during follow-up versus those who did not). For such event-based dichotomous data, we used RR or OR (with 95% CIs) as effect sizes, which is a standard statistical approach for quantifying associations between exposures and binary outcomes in observational studies.

The I² statistic is a core indicator for assessing heterogeneity among studies, with a range of 0% to 100%. Currently, the most commonly used international standard is the interpretation guidelines for I² values proposed by Higgins et al. (2022) (42). Heterogeneity among studies for each association was tested, with results expressed as *P*-values and I² values. If *P* > 0.1 and I² < 50%, this indicates low heterogeneity, and a fixed-effects model is used for analysis; otherwise, a random-effects model is applied. Egger’s test was used to evaluate publication bias among the included studies. P < 0.05 indicates statistically significant publication bias.

## 3 Results

### 3.1 Article screening process

A total of 7,274 articles were obtained from the initial screening. We followed a systematic, cascading exclusion-based study screening process and finally included 7 articles. Publications of the included studies spanned from 2008 to 2024.

**Figure 1:**
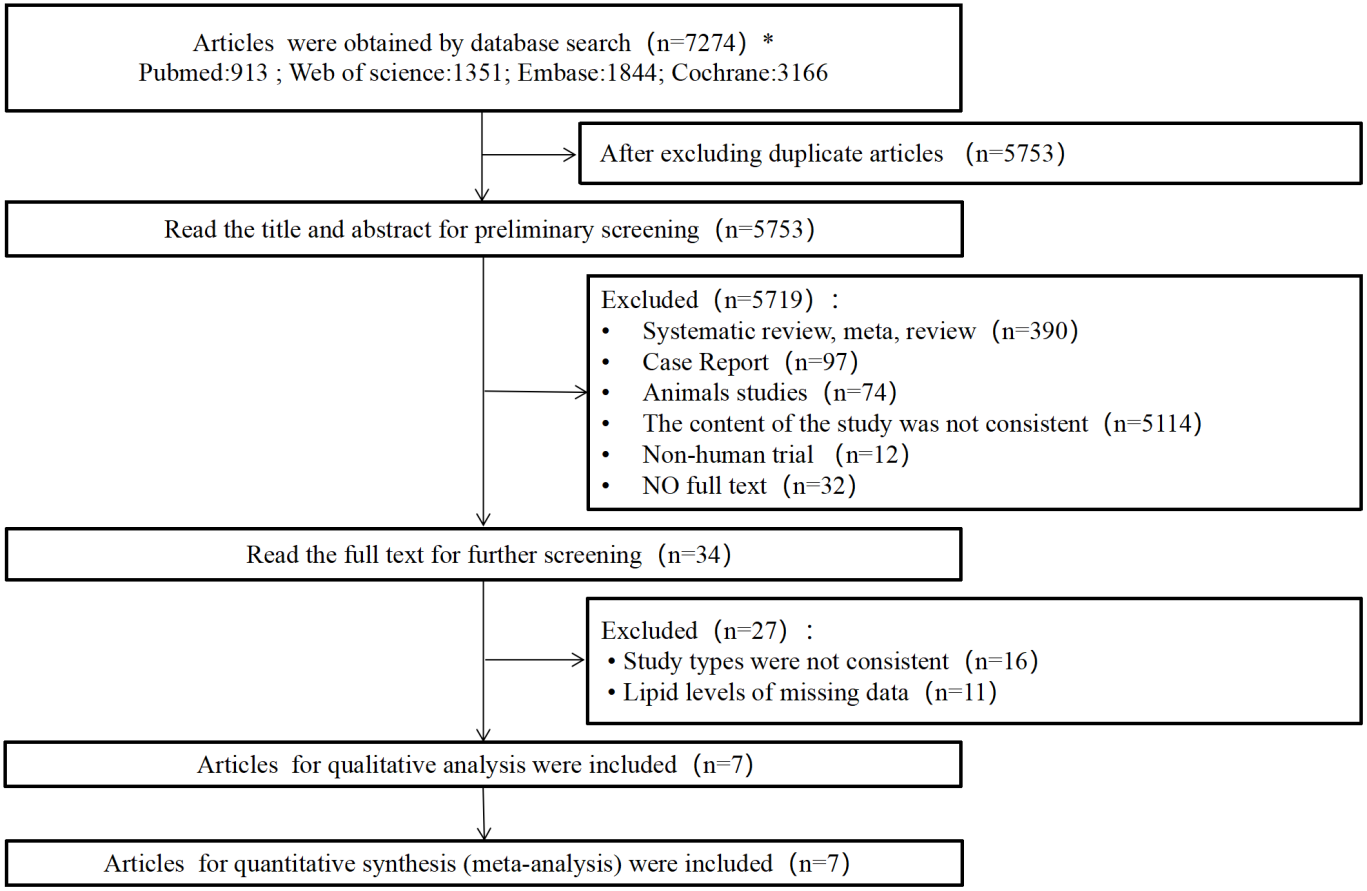
Article screening flowchart.

Of these 7 included studies, four were carried out in France (43–46), one in China (47), one in Canada (48), and one in Tel Aviv, Israel (49). A PRISMA-compliant screening flow chart is shown in **Figure 1**.

### 3.2 Basic characteristics of the included studies

The basic characteristics of the included articles are shown in **Table 1**, including 7 articles with a total of 17,465 participants. Five studies (43–45, 47, 48) reported the effect of depressive disorders on blood lipids, and two studies (46, 49) reported the effect of blood lipids on the onset of depression. All of the studies were cohort studies, and the quality of the included articles was evaluated by the NOS; the overall scores ranged from 6 to 8, which were relatively good, as shown in **Table 2**.

**Table 1:** Basic characteristics of the included studies.

Type	Author	Year	Country	Study type	Sample source	Age	Sample size	Male/female	Index	Influence Factor	Follow-up
Depression → Lipid	Ancelin ML	2010	France	longitudinal study	Montpellier district	65+	1792	752/1040	HDL/LDL/TG/TC	age/education	7-year follow-up
Depression → Lipid	Akbaraly TN	2011	France	cohort study	electoral rolls	–	4446	–	HDL/TG/TC	Age/sex.etc	2- and 4-year follow-ups
Depression → Lipid	El Asmar K	2023	France	Cohort Study	six university psychiatry departments	42.3 ± 13.0	117	38/83	HDL/TG	Age/Sex.etc	3 and 6 months
Depression → Lipid	Khalfan AF	2023	Canada	Cohort Study	children’s hospital	15.0 ± 1.9	239	–	HDL	Sex/age/BMI	–
Depression → Lipid	Cheng P	2024	China	Cohort Study	the Second Xiangya Hospital of Central South University	26.4 ± 15.31	1429	513/943	HDL/LDL	Region of residence/Thyroid stimulating hormone	spanning 90, 180, and 365 days
Lipid → Depression	Toker S	2008	Tel Aviv	logistic regression analysis	healthy employees	45.2 ± 10.6/46.2 ± 10.1	3880	2355/1525	HDL/TG	–	2 years
Lipid → Depression	Akbaraly TN	2009	London	cohort study	office staff	41-61	5562	3958/1604	HDL/LDL/TG	Sex/age. etc	After 6 years

**Table 2:** Quality evaluation of included studies.

Study (cohort)	Representative of the exposed cohort	Selection of non-exposed cohort	Ascertainment of exposure	Outcome not present before study	Comparability	Assessment of outcome	Follow-up long enough	Adequacy of follow up	Quality score
Toker S 2008	*	*	*	*	*	*	*		7
Akbaraly TN 2009	*	*	*	*	*	*	*		7
Ancelin ML 2010	*	*	*		*	*	*	*	7
Akbaraly TN 2011	*	*	*	*	*	*	*	*	8
El Asmar K 2023	*		*	*		*	*	*	6
Khalfan AF 2023	*	*	*	*	*	*			6
Cheng P 2024	*	*	*		*	*	*		6

## 4 Analysis of results

### 4.1 Effect of lipid parameters on the onset of depression

**Figure 2:**
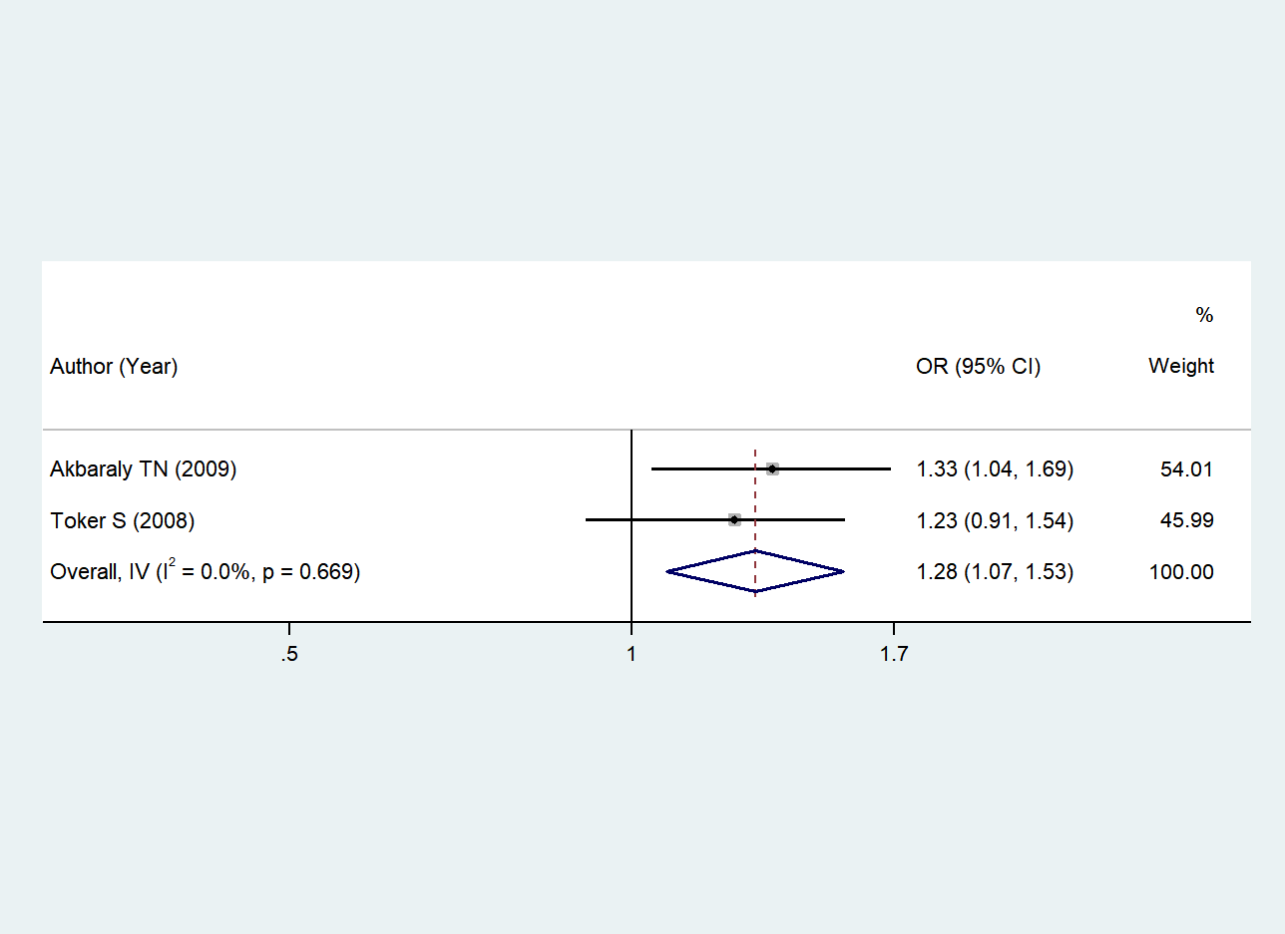
The association between HDL and depression. Each horizontal line segment in the figure represents the 95% confidence interval of the corresponding effect size.

Two studies (46, 49) reported the association between serum HDL levels and depression. A fixed-effects model was chosen due to low heterogeneity (I² = 0%, *P* = 0.669). Meta-analysis based on this model showed that changes in serum HDL levels were associated with an increased risk of depression (OR = 1.28, 95% CI: 1.07–1.53), indicating that individuals with abnormal serum HDL levels had a 28% higher risk of depression compared to those with normal levels. The *Z*-statistic for the overall effect was 2.738, with *P* = 0.006 < 0.05, indicating statistically significant results, as shown in **Figure 2** (Forest plot of the association between serum HDL levels and depression).

Only one study (46) reported the association between changes in LDL levels and depression. The results showed that changes in LDL levels were not significantly associated with the risk of depression (OR = 1.26, 95% CI: 0.98–1.61; *Z* = 1.825, *P* = 0.068 > 0.05).

**Figure 3:**
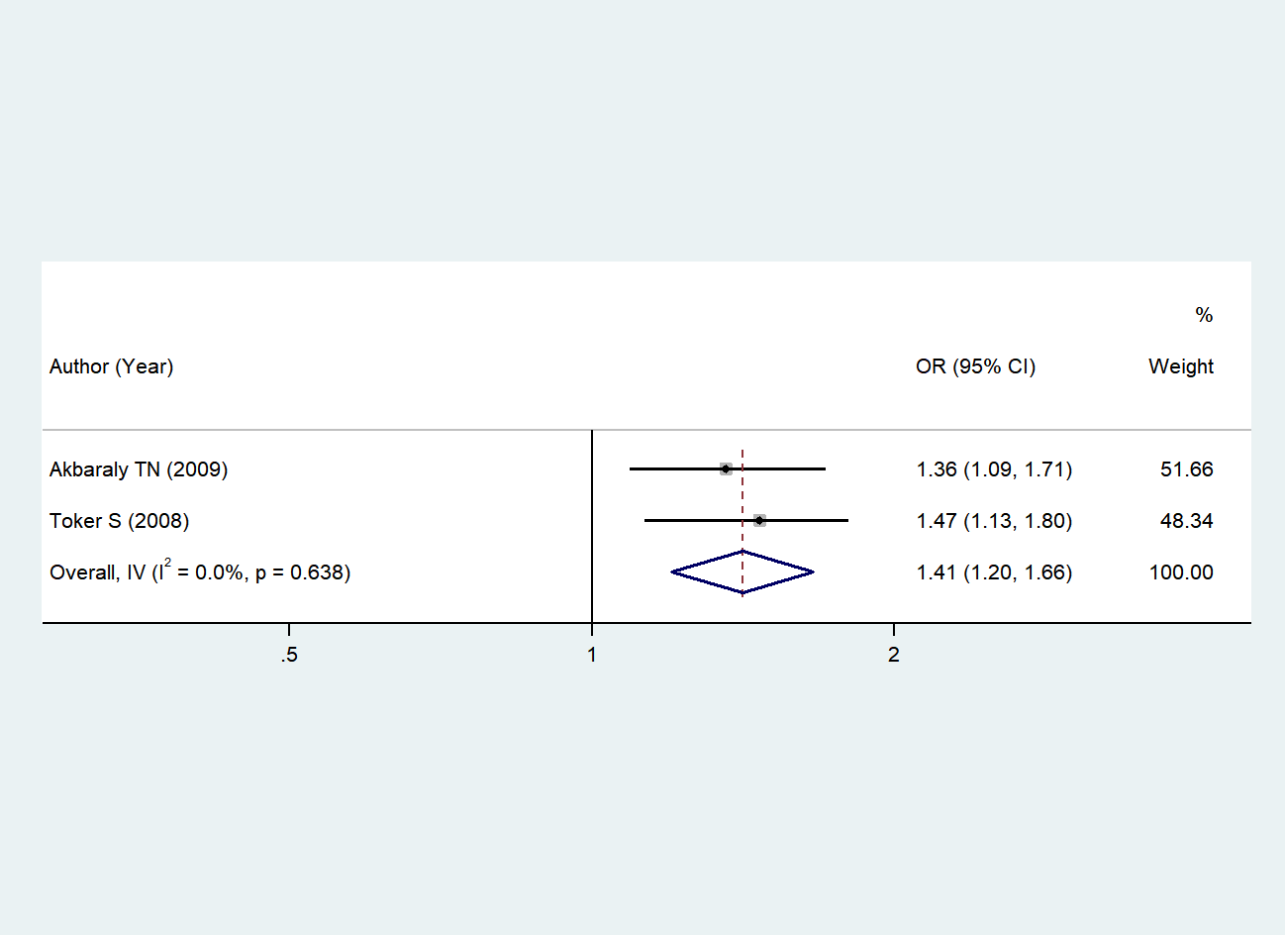
The association between triglycerides and depression.

Two studies (46, 49) reported the association between serum triglyceride levels and depression. Due to low heterogeneity (I² = 0%, *P* = 0.638), a fixed-effects model was chosen. Meta-analysis based on this model showed that changes in serum triglyceride levels were significantly associated with a higher risk of depression; the pooled overall effect was OR = 1.41, 95% CI: 1.20–1.66 (*Z* = 4.179, *P* < 0.001). These statistically significant results are shown in **Figure 3** (Forest plot of the association between serum triglyceride levels and depression).

### 4.2 Effect of depression on lipid levels

**Figure 4:**
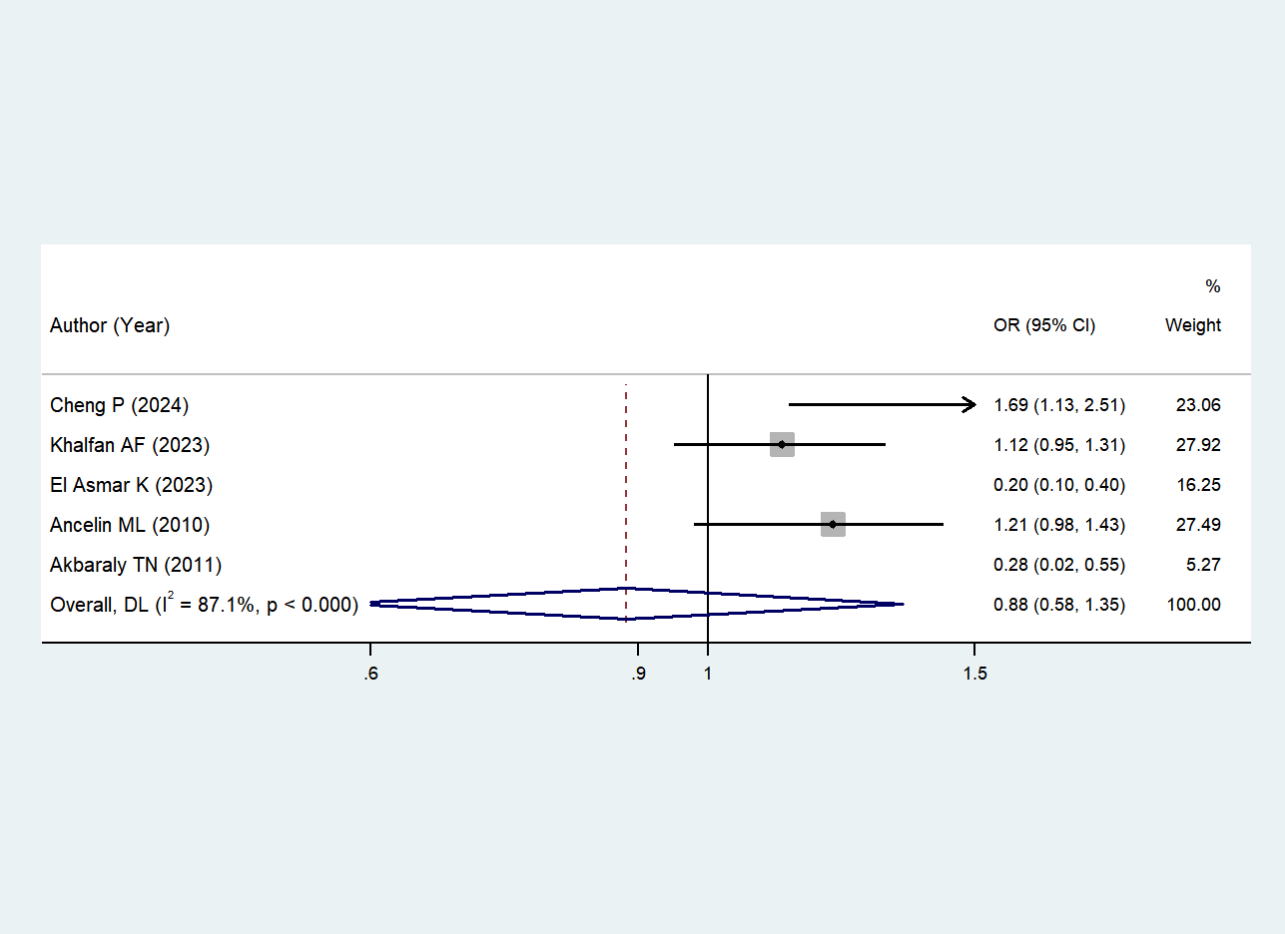
The effect of depression on HDL.

Five studies (43–45, 47, 48) reported the association between depression and serum HDL levels. Due to high heterogeneity (I² = 87.1%, *P* < 0.001), a random-effects model was chosen; potential sources of heterogeneity will be discussed in the subsequent section. The results showed that depression was not significantly associated with changes in serum HDL levels when comparing depressed individuals with non-depressed individuals (OR = 0.88, 95% CI: 0.58–1.35; *Z* = -0.578, *P* = 0.563 > 0.05). See **Figure 4** (Forest plot of the association between depression and serum HDL levels) for details.

### 4.3 Subgroup analysis

Due to high heterogeneity (I² = 87.1%, P < 0.001) in the five studies investigating the association between depressive symptoms and serum HDL levels, we performed a subgroup analysis according to the countries where the studies were carried out. Subgroup-specific heterogeneity and statistical results (OR, 95% CI, *Z*, *P*) were as follows: France (43–45) I² = 92.5%, (high heterogeneity), (*Z* = -1.120, *P* = 0.263 > 0.05, no statistical significance); Canada (48) Single study, no heterogeneity, (*Z* = 1.383, *P* = 0.167 > 0.05, no statistical significance); and China (47) Single study, no heterogeneity, (*Z* = 2.569, *P* = 0.010 < 0.05, statistically significant).

**Figure 5:**
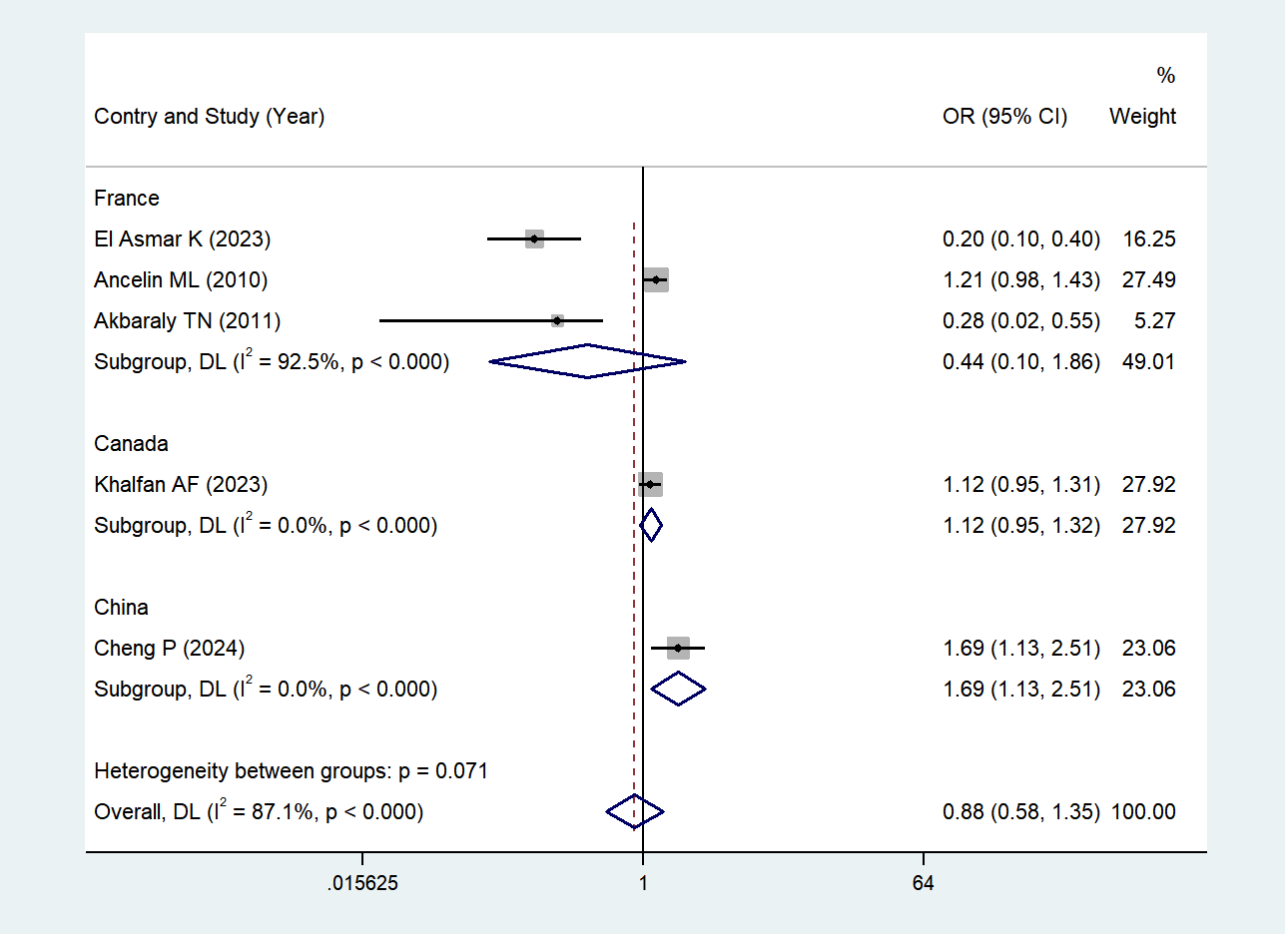
Based on the results of subgroup analyses conducted in countries.

We found that in the Chinese study (47), there was a significant negative association between depressive symptoms and serum HDL levels: individuals with depressive symptoms had significantly lower serum HDL levels than non-depressed individuals, and this association was statistically significant. See **Figure 5** (Subgroup analysis forest plot for the association between depressive symptoms and serum HDL levels in China) for details.

**Figure 6:**
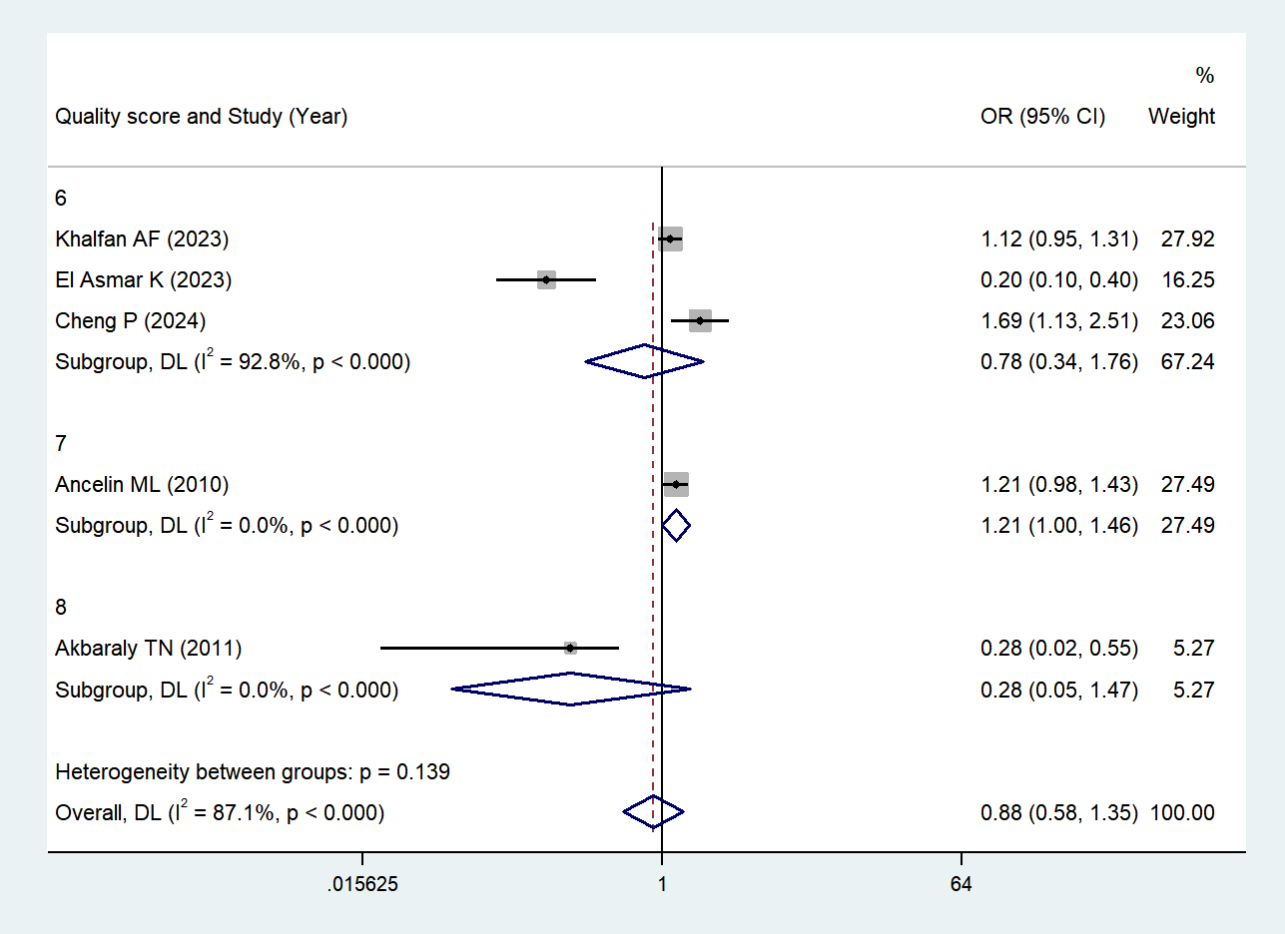
Subgroup analyses based on study quality scores.

We further performed a subgroup analysis based on study quality scores: For studies with a score of 6 (45, 48): *Z* = -0.604, *P* = 0.546 > 0.05; For studies with a score of 7 (44): *Z* = 1.977, *P* = 0.048 < 0.05; For studies with a score of 8 (43): *Z* = -1.506, *P* = 0.132 > 0.05. We found that in studies with a score of 7 (44), the association between depressive symptoms and serum HDL levels was stronger than that in non-depressed individuals, with a statistically significant difference. See **Figure 6** (Subgroup analyses based on study quality scores) for details.

**Figure 7:**
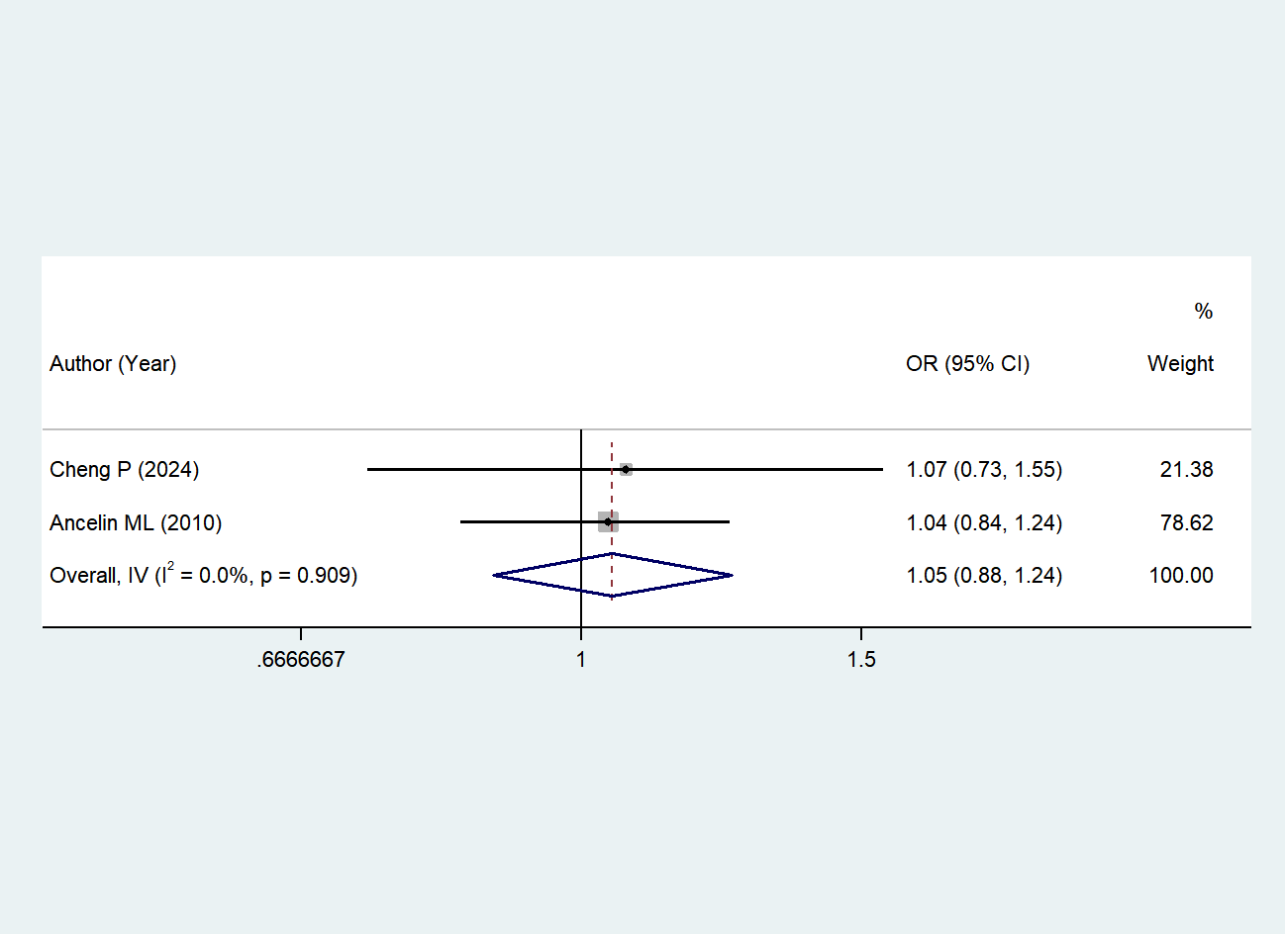
The effect of depression on LDL.

The association between depression and serum LDL levels was reported in 2 studies (44, 47). Due to low heterogeneity (I² = 0%, *P* = 0.909), a fixed-effects model was chosen. The results showed that the association between depressive disorders and serum LDL levels was not statistically significant when compared with non-depressed individuals (OR = 1.05, 95% CI: 0.88–1.24; *Z* = 0.505, *P* = 0.613 > 0.05). See **Figure 7**.

**Figure 8:**
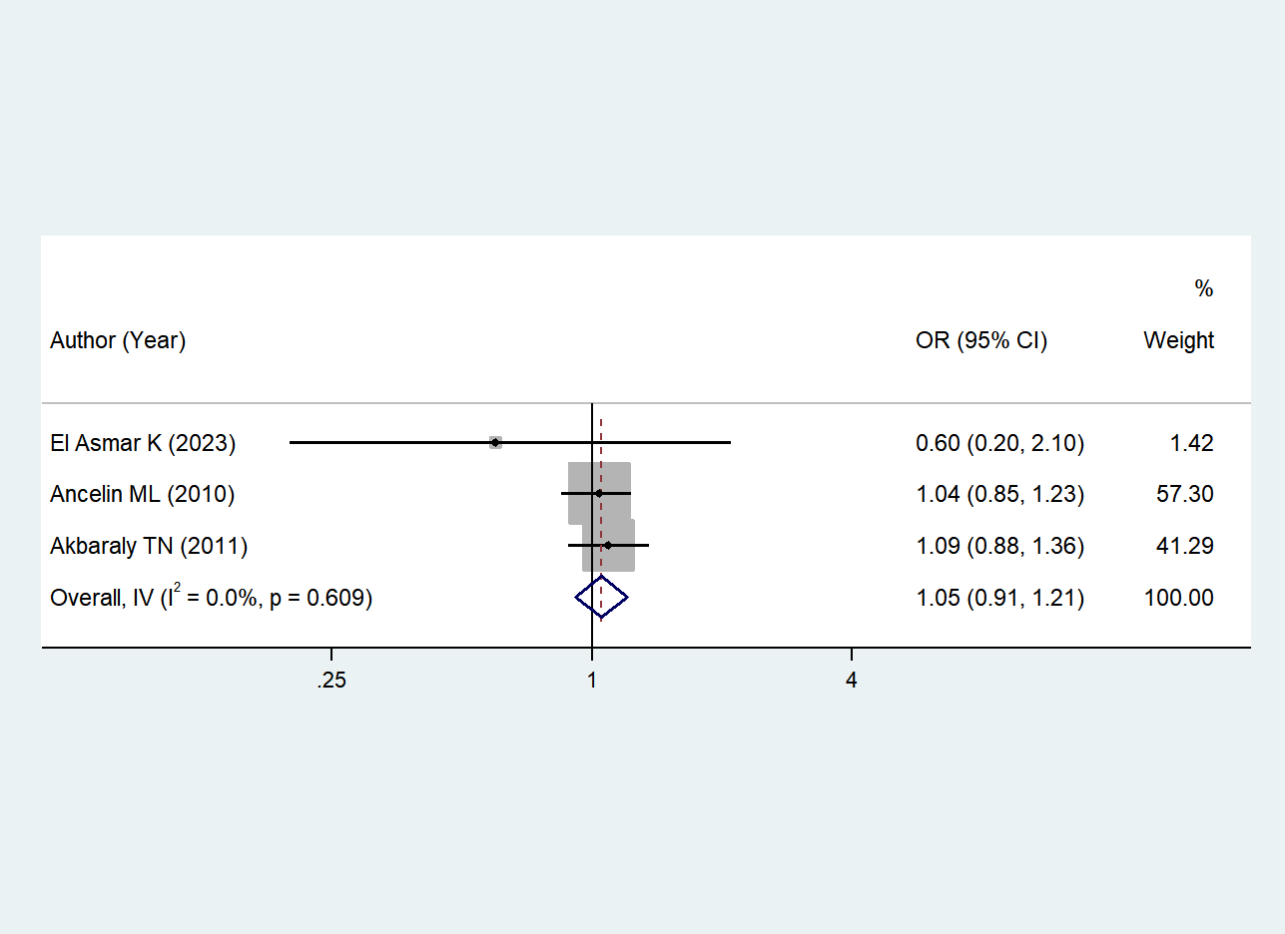
The effect of depressive symptoms on serum triglycerides.

The association between depressive symptoms and serum triglyceride levels was reported in three studies (43–45). Due to low heterogeneity (I² = 0%, *P* = 0.609), a fixed-effects model was chosen. The results showed that the association between depressive symptoms and serum triglyceride levels was not statistically significant when compared with non-depressed individuals (OR = 1.05, 95% CI: 0.91–1.21; *Z* = 0.712, *P* = 0.476 > 0.05). See **Figure 8**.

Only one study (44) reported the association between depression and serum total cholesterol levels. The results showed that the association between depressive symptoms and serum total cholesterol levels was not statistically significant when compared with non-depressed individuals (OR = 1.11, 95% CI: 0.90–1.32), with *Z* = 1.068 and *P* = 0.285 > 0.05.

### 4.4 Publication bias analysis

**Figure 9:**
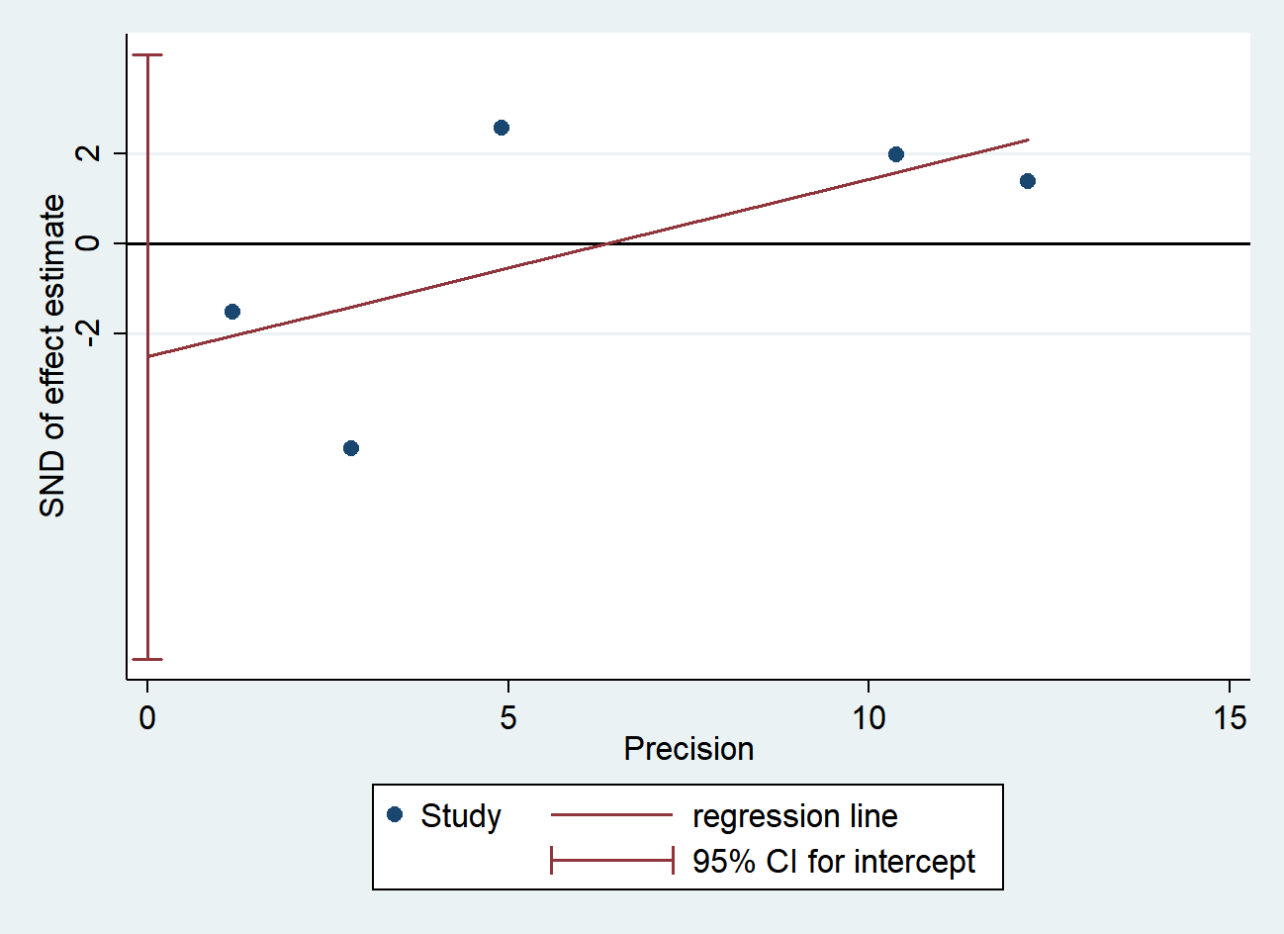
Publication bias analysis.

Egger’s test was used to assess publication bias associated with the effect of depressive symptoms on serum HDL levels. The results were visualized using a funnel plot (**Figure 9**), which showed a relatively symmetric distribution. Egger’s test yielded *P* = 0.320 > 0.05, collectively indicating no significant publication bias.

## 5 Discussion

The main objective of this meta-analysis was to systematically analyze the bidirectional and causal relationships between lipid parameters and depression: specifically, to investigate whether dyslipidemia exacerbates depression severity, whether depression further affects lipid levels, and whether these clinical indicators can serve as biomarkers for assessing depression risk. By analyzing seven included studies, we found that changes in serum HDL and triglyceride levels were significantly associated with an increased risk of depression (HDL: OR = 1.28, 95% CI: 1.07–1.53; triglycerides: OR = 1.41, 95% CI: 1.20–1.66; both *P* < 0.05). In contrast, compared with non-depressed individuals, depressive disorders were not significantly associated with changes in serum HDL, LDL, triglyceride, or total cholesterol levels (HDL: OR = 0.88, 95% CI: 0.58–1.35; LDL: OR = 1.05, 95% CI: 0.88–1.24; triglycerides: OR = 1.05, 95% CI: 0.91–1.21; total cholesterol: OR = 1.11, 95% CI: 0.90–1.32; all *P* > 0.05).

In summary, the risk of depression is associated with changes in serum HDL and triglyceride levels, and these lipid indicators can serve as biomarkers for assessing depression risk. The pathogenesis of depression is complex, involving biological, psychological, and social environmental factors. This study focuses on the bidirectional relationship between blood lipids and depression; investigating this association is of great significance for understanding the pathogenesis of depression, formulating prevention strategies, and developing individualized treatment plans for patients.

At the same time, this study can help clinicians evaluate the lipid status of patients with depression, develop targeted and timely treatment plans, and provide guidance for family members on patient care—such as instructing patients to adhere to a diet rich in dietary fiber and low in saturated fat, and engage in moderate-intensity physical activity to manage lipid levels. Ultimately, this may help minimize the exacerbation or recurrence of depression in affected patients through lipid control.

For clinical practice, the following specific recommendations are provided: Clinicians are recommended to perform a four-component lipid profile (total cholesterol, triglycerides, LDL-C, HDL-C) and apolipoprotein A1/B testing in patients with depression during their first visit. This is particularly important for patients with obesity, metabolic syndrome, or those receiving long-term antidepressant therapy—some of which may affect lipid metabolism. For these high-risk patients, lipid testing should be repeated every 3–6 months to dynamically monitor changes in lipid metabolism.

If a depressed patient presents with abnormal lipid parameters and moderate-to-severe depressive symptoms, it is recommended to consult with an endocrinologist first. Based on the consultation advice, psychological intervention, antidepressant treatment, and lipid-lowering intervention can be combined: start with lifestyle modifications; if lipid levels remain abnormal after 3 months, short-term lipid-lowering medications may be initiated—with close monitoring of potential adverse effects and drug-drug interactions with antidepressants.

## 6 Limitation of the study

Egger’s test results indicated no significant publication bias among the included studies, suggesting that the results of this meta-analysis are relatively reliable—at least in terms of publication bias control. However, several limitations should be acknowledged: First, the included studies were not strictly categorized by key variables, and most lacked explicit subgrouping. This inconsistency may have introduced some selection bias into the results. Second, the scales used to assess depression in the included studies were not standardized, which may have compromised the accuracy of outcome assessment. Third, the number of included studies investigating the association between lipid parameters and depression was relatively small. Additionally, many relevant studies only assessed whether overall lipid levels were abnormal and did not report detailed lipid parameters, so these studies could not be included in this meta-analysis. Furthermore, the search was limited to English-language publications during the literature retrieval process, which may exclude region-specific evidence published in other languages. Therefore, future studies should include non-English literature to deepen our understanding of this association.

The articles included in this study cover regions of France (n = 4 studies), China (n = 1 study), Canada (n = 1 study), and Tel Aviv, Israel (n = 1 study). This uneven distribution might affect the generalizability of result interpretation. First, interference from regional and dietary cultural differences: The included studies were from France (with a Mediterranean diet pattern, characterized by high intake of unsaturated fats and dietary fiber), China (with an East Asian diet pattern, characterized by high carbohydrate intake and low saturated fat intake), Canada (with a Western diet pattern, high in processed foods and saturated fat), and Tel Aviv, Israel (with a Middle Eastern diet pattern, high in olive oil and spice intake). Varied dietary patterns have regulatory effects on lipid metabolism, and these regional and dietary cultural differences may lead to variations in the magnitude of the association between lipid levels, 5-HT, and depression across studies—for example, the high unsaturated fat intake in the Mediterranean diet may mitigate the negative association between low HDL and depression, resulting in a weaker effect size in French studies (50, 51). Second, the potential influence of genetic background: The included studies covered the Caucasian population (from France, Canada, and Tel Aviv, Israel) and the East Asian population (from China). Different populations vary in the allele frequencies of genes related to lipid metabolism and key rate-limiting enzymes for 5-HT synthesis. These genetic differences may lead to population-specific sensitivity thresholds for the effects of lipids and 5-HT on depression, thereby increasing heterogeneity among studies. Therefore, future research directions should consider the following improvements: use subgroup analysis (stratified by region, population, and dietary pattern) or meta-regression analysis (with “diet type” and “population” as covariates) to quantify the impact of heterogeneity sources on the association effect and improve the accuracy of result interpretation.

Finally, although serum 5-HT was included in our search strategy, we did not identify any studies that met the inclusion criteria. The missing 5-HT data may lead to two limitations: First, although this study revealed the bidirectional association between lipid indicators and depression, the lack of 5-HT data prevented us from conducting mediation analysis to verify whether lipids regulate serum 5-HT levels to indirectly affect the onset of depression—thus failing to clarify the key mediating pathway. Second, there is a potential impact on the applicability of the study results. Some patients with depression may have abnormal serum 5-HT levels independently of lipids (e.g., primary 5-HT synthesis disorder). Due to the lack of this indicator data, this study cannot distinguish subgroup differences between “pure lipid-related depression” (depression with normal serum 5-HT levels) and “depression related to both lipids and 5-HT” (depression with concurrent lipid abnormalities and 5-HT dysregulation). This may reduce the applicability of the study conclusions to patients with comorbid abnormal 5-HT levels and also fails to provide a reference for whether it is necessary to monitor 5-HT in conjunction with lipid indicators to optimize depression risk stratification in clinical practice.

## 7 Conclusion

In summary, our meta-analysis shows that among serum lipid parameters, lower HDL levels and higher triglyceride levels exhibit a stronger association with depression onset. This relationship suggests that integrating these lipid indices could aid early intervention, potentially improving prognosis and quality of life in depressed patients. Despite this potential, current evidence remains insufficient. Further large-scale, prospective studies—particularly those focusing on clinical utility, determination of cutoff values, and validation in specific populations—are needed before these biomarkers can be widely implemented in clinical practice.

## Data Availability

The original contributions presented in the study are included in the article/supplementary material. Further inquiries can be directed to the corresponding authors.
