# Supplementary material for: Exploring the bidirectional relationship between depressive disorder and dyslipidemia: a systematic review and meta-analysis
Source: Front Psychiatry. 2025 Dec 10;16:1498773. doi: 10.3389/fpsyt.2025.1498773 (PMC12728578; doi:10.3389/fpsyt.2025.1498773)
Supplement: Supplementary file 1 [file Table1.doc]

**Retrieval procedure**

**Cochrane：3166**

Search Name:

Date Run: 04/05/2024 12:01:18

Comment:

ID Search Hits

#1 MeSH descriptor: [Depressive Disorder] explode all trees 16619

#2 (Depressive Disorder OR Depressive Disorders OR Disorder, Depressive OR Disorders, Depressive OR Neurosis, Depressive OR Depressive Neuroses OR Depressive Neurosis OR Neuroses, Depressive OR Depression, Endogenous OR Depressions, Endogenous OR Endogenous Depression OR Endogenous Depressions OR Depressive Syndrome OR Depressive Syndromes OR Syndrome, Depressive OR Syndromes, Depressive OR Depression, Neurotic OR Depressions, Neurotic OR Neurotic Depression OR Neurotic Depressions OR Melancholia OR Melancholias OR Unipolar Depression OR Depression, Unipolar OR Depressions, Unipolar OR Unipolar Depressions):ti,ab,kw (Word variations have been searched) 58221

#3 #1 OR #2 58829

#4 MeSH descriptor: [Serotonin] explode all trees 1361

#5 (Serotonin OR Hippophaine OR 3 (2 Aminoethyl) 1H indol 5 ol OR Enteramine OR 5 HT OR 5 Hydroxytryptamine OR 5 Hydroxytryptamine OR Hydroxytryptamine OR Epicholesterol):ti,ab,kw (Word variations have been searched) 15062

#6 #4 OR #5 15066

#7 MeSH descriptor: [Cholesterol] explode all trees 13097

#8 MeSH descriptor: [Cholesterol, HDL] explode all trees 4622

#9 (HDL聽Lipoproteins OR High-Density Lipoprotein OR Lipoprotein, High-Density OR High-Density Lipoproteins OR High Density Lipoproteins OR Lipoproteins, High-Density OR alpha-Lipoproteins OR alpha Lipoproteins OR Heavy Lipoproteins OR Lipoproteins, Heavy OR High Density Lipoprotein OR Density Lipoprotein, High OR Lipoprotein, High Density OR alpha-Lipoprotein OR alpha Lipoprotein OR alpha 1 Lipoprotein):ti,ab,kw 17683

#10 #8 OR #9 19356

#11 MeSH descriptor: [Triglycerides] explode all trees 8014

#12 (Triacylglycerols OR Triacylglycerol OR Triglyceride):ti,ab,kw 17478

#13 #11 OR #12 21308

#14 MeSH descriptor: [Cholesterol, LDL] explode all trees 6202

#15 (Low Density Lipoprotein Cholesterol OR beta-Lipoprotein Cholesterol OR Cholesterol, beta-Lipoprotein OR beta Lipoprotein Cholesterol OR LDL聽Cholesterol OR Cholesteryl Linoleate,聽LDL OR LDL聽Cholesteryl Linoleate):ti,ab,kw 27026

#16 #14 OR #15 27026

#17 MeSH descriptor: [Cholesterol, VLDL] explode all trees 287

#18 (VLDL Cholesterol OR Pre-beta-Lipoprotein Cholesterol OR Cholesterol, Pre-beta-Lipoprotein OR Pre beta Lipoprotein Cholesterol OR Very Low Density Lipoprotein Cholesterol OR Prebetalipoprotein Cholesterol OR Cholesterol, Prebetalipoprotein):ti,ab,kw 3101

#19 #17 OR #18 3101

#20 #6 OR #7 OR #10 OR #13 OR #16 OR 19 283729

#21 MeSH descriptor: [Longitudinal Studies] explode all trees 8787

#22 (prospective OR follow-up OR followup):ti,ab,kw 521662

#23 #21 OR #22 526367

#24 #3 AND #20 AND #23 3166

**Embase：1844**

Embase

Session Results

.......................................................

No. Query Results Results Date

#26. #3 AND #22 AND #25 1,844 4 May 2024

#25. #23 OR #24 3,330,912 4 May 2024

#24. 'prospective':ab,ti OR 'follow-up':ab,ti OR 2,952,383 4 May 2024

'followup':ab,ti

#23. longitudinal 529,812 4 May 2024

#22. #6 OR #9 OR #12 OR #15 OR #18 OR #21 876,804 4 May 2024

#21. #19 OR #20 35,930 4 May 2024

#20. 'vldl cholesterol':ab,ti OR 'pre-beta-lipoprotein 4,136 4 May 2024

cholesterol':ab,ti OR 'cholesterol,

pre-beta-lipoprotein':ab,ti OR 'pre beta

lipoprotein cholesterol':ab,ti OR 'very low

density lipoprotein cholesterol':ab,ti OR

'prebetalipoprotein cholesterol':ab,ti OR

'cholesterol, prebetalipoprotein':ab,ti

#19. very AND low AND density AND lipoprotein AND 35,512 4 May 2024

cholesterol

#18. #16 OR #17 232,621 4 May 2024

#17. 'low density lipoprotein cholesterol':ab,ti OR 117,360 4 May 2024

'beta-lipoprotein cholesterol':ab,ti OR

'cholesterol, beta-lipoprotein':ab,ti OR 'beta

lipoprotein cholesterol':ab,ti OR

'ldl聽cholesterol':ab,ti OR 'cholesteryl

linoleate,聽ldl':ab,ti OR 'ldl聽cholesteryl

linoleate':ab,ti

#16. low AND density AND lipoprotein AND cholesterol 224,436 4 May 2024

#15. #13 OR #14 125,486 4 May 2024

#14. 'triacylglycerols':ab,ti OR 122,583 4 May 2024

'triacylglycerol':ab,ti OR 'triglyceride':ab,ti

#13. triglyceride 104,976 4 May 2024

#12. #10 OR #11 103,621 4 May 2024

#11. 'hdl聽lipoproteins':ab,ti OR 'high-density 87,993 4 May 2024

lipoprotein':ab,ti OR 'lipoprotein,

high-density':ab,ti OR 'high-density

lipoproteins':ab,ti OR 'high density

lipoproteins':ab,ti OR 'lipoproteins,

high-density':ab,ti OR 'alpha-lipoproteins':ab,ti

OR 'heavy lipoproteins':ab,ti OR 'lipoproteins,

heavy':ab,ti OR 'high density lipoprotein':ab,ti

OR 'density lipoprotein, high':ab,ti OR

'lipoprotein, high density':ab,ti OR

'alpha-lipoprotein':ab,ti OR 'alpha

lipoprotein':ab,ti OR 'alpha-1 lipoprotein':ab,ti

#10. 'hdl c' 35,987 4 May 2024

#9. #7 OR #8 553,596 4 May 2024

#8. 'epicholesterol':ab,ti 88 4 May 2024

#7. cholesterol 553,594 4 May 2024

#6. #4 OR #5 257,219 4 May 2024

#5. 'serotonin':ab,ti OR 'hippophaine':ab,ti OR 163,857 4 May 2024

'3-(2-aminoethyl)-1h-indol-5-ol':ab,ti OR

'enteramine':ab,ti OR '5-ht':ab,ti OR

'5-hydroxytryptamine':ab,ti OR '5

hydroxytryptamine':ab,ti OR

'hydroxytryptamine':ab,ti

#4. serotonin 250,771 4 May 2024

#3. #1 OR #2 130,734 4 May 2024

#2. 'depressive disorder':ab,ti OR 'depressive 77,588 4 May 2024

disorders':ab,ti OR 'disorder, depressive':ab,ti

OR 'disorders, depressive':ab,ti OR 'neurosis,

depressive':ab,ti OR 'depressive neuroses':ab,ti

OR 'depressive neurosis':ab,ti OR 'neuroses,

depressive':ab,ti OR 'depression,

endogenous':ab,ti OR 'depressions,

endogenous':ab,ti OR 'endogenous

depression':ab,ti OR 'endogenous

depressions':ab,ti OR 'depressive syndrome':ab,ti

OR 'depressive syndromes':ab,ti OR 'syndrome,

depressive':ab,ti OR 'syndromes,

depressive':ab,ti OR 'depression, neurotic':ab,ti

OR 'depressions, neurotic':ab,ti OR 'neurotic

depression':ab,ti OR 'neurotic depressions':ab,ti

OR 'melancholia':ab,ti OR 'melancholias':ab,ti OR

'unipolar depression':ab,ti OR 'depression,

unipolar':ab,ti OR 'depressions, unipolar':ab,ti

OR 'unipolar depressions':ab,ti

#1. depressive AND disorder 115,960 4 May 2024

.......................................................

**Pubmed: 913**

Search number Query Sort By Filters Search Details Results Time

29 "(((""Depressive Disorder""[Mesh]) OR ((((((((((((((((((((((((((Depressive Disorder[Title/Abstract]) OR (Depressive Disorders[Title/Abstract])) OR (Disorder, Depressive[Title/Abstract])) OR (Disorders, Depressive[Title/Abstract])) OR (Neurosis, Depressive[Title/Abstract])) OR (Depressive Neuroses[Title/Abstract])) OR (Depressive Neurosis[Title/Abstract])) OR (Neuroses, Depressive[Title/Abstract])) OR (Depression, Endogenous[Title/Abstract])) OR (Depressions, Endogenous[Title/Abstract])) OR (Endogenous Depression[Title/Abstract])) OR (Endogenous Depressions[Title/Abstract])) OR (Depressive Syndrome[Title/Abstract])) OR (Depressive Syndromes[Title/Abstract])) OR (Syndrome, Depressive[Title/Abstract])) OR (Syndromes, Depressive[Title/Abstract])) OR (Depression, Neurotic[Title/Abstract])) OR (Depressions, Neurotic[Title/Abstract])) OR (Neurotic Depression[Title/Abstract])) OR (Neurotic Depressions[Title/Abstract])) OR (Melancholia[Title/Abstract])) OR (Melancholias[Title/Abstract])) OR (Unipolar Depression[Title/Abstract])) OR (Depression, Unipolar[Title/Abstract])) OR (Depressions, Unipolar[Title/Abstract])) OR (Unipolar Depressions[Title/Abstract]))) AND (((((""Serotonin""[Mesh]) OR ((((((((Serotonin[Title/Abstract]) OR (Hippophaine[Title/Abstract])) OR (3-(2-Aminoethyl)-1H-indol-5-ol[Title/Abstract])) OR (Enteramine[Title/Abstract])) OR (5-HT[Title/Abstract])) OR (5-Hydroxytryptamine[Title/Abstract])) OR (5 Hydroxytryptamine[Title/Abstract])) OR (Hydroxytryptamine[Title/Abstract]))) OR ((""Lipoproteins, HDL""[Mesh]) OR ((((((((((((((((HDL Lipoproteins[Title/Abstract]) OR (High-Density Lipoprotein[Title/Abstract])) OR (Lipoprotein, High-Density[Title/Abstract])) OR (High-Density Lipoproteins[Title/Abstract])) OR (High Density Lipoproteins[Title/Abstract])) OR (Lipoproteins, High-Density[Title/Abstract])) OR (alpha-Lipoproteins[Title/Abstract])) OR (alpha-Lipoproteins[Title/Abstract])) OR (Heavy Lipoproteins[Title/Abstract])) OR (Lipoproteins, Heavy[Title/Abstract])) OR (High Density Lipoprotein[Title/Abstract])) OR (Density Lipoprotein, High[Title/Abstract])) OR (Lipoprotein, High Density[Title/Abstract])) OR (alpha-Lipoprotein[Title/Abstract])) OR (alpha Lipoprotein[Title/Abstract])) OR (alpha-1 Lipoprotein[Title/Abstract])))) OR ((""Cholesterol, LDL""[Mesh]) OR (((((((Low Density Lipoprotein Cholesterol[Title/Abstract]) OR (beta-Lipoprotein Cholesterol[Title/Abstract])) OR (Cholesterol, beta-Lipoprotein[Title/Abstract])) OR (beta Lipoprotein Cholesterol[Title/Abstract])) OR (LDL Cholesterol[Title/Abstract])) OR (Cholesteryl Linoleate, LDL[Title/Abstract])) OR (LDL Cholesteryl Linoleate[Title/Abstract])))) OR ((""Cholesterol, VLDL""[Mesh]) OR (((((((VLDL Cholesterol[Title/Abstract]) OR (Pre-beta-Lipoprotein Cholesterol[Title/Abstract])) OR (Cholesterol, Pre-beta-Lipoprotein[Title/Abstract])) OR (Pre beta Lipoprotein Cholesterol[Title/Abstract])) OR (Very Low Density Lipoprotein Cholesterol[Title/Abstract])) OR (Prebetalipoprotein Cholesterol[Title/Abstract])) OR (Cholesterol, Prebetalipoprotein[Title/Abstract]))))) AND ((""Longitudinal Studies""[Mesh]) OR ((((longitudinal[Title/Abstract]) OR (prospective[Title/Abstract])) OR (follow-up[Title/Abstract])) OR (followup[Title/Abstract])))" "(""Depressive Disorder""[MeSH Terms] OR (""Depressive Disorder""[Title/Abstract] OR ""depressive disorders""[Title/Abstract] OR ""disorder depressive""[Title/Abstract] OR ""disorders depressive""[Title/Abstract] OR ""neurosis depressive""[Title/Abstract] OR ""depressive neuroses""[Title/Abstract] OR ""depressive neurosis""[Title/Abstract] OR ""neuroses depressive""[Title/Abstract] OR ""depression endogenous""[Title/Abstract] OR ""depressions endogenous""[Title/Abstract] OR ""endogenous depression""[Title/Abstract] OR ""endogenous depressions""[Title/Abstract] OR ""depressive syndrome""[Title/Abstract] OR ""depressive syndromes""[Title/Abstract] OR ""syndrome depressive""[Title/Abstract] OR ""syndromes depressive""[Title/Abstract] OR ""depression neurotic""[Title/Abstract] OR ((""depressed""[Title/Abstract] OR ""Depression""[MeSH Terms] OR ""Depression""[Title/Abstract] OR ""Depressions""[Title/Abstract] OR ""depression s""[Title/Abstract] OR ""Depressive Disorder""[MeSH Terms] OR (""Depressive""[Title/Abstract] AND ""Disorder""[Title/Abstract]) OR ""Depressive Disorder""[Title/Abstract] OR ""depressivity""[Title/Abstract] OR ""Depressive""[Title/Abstract] OR ""depressively""[Title/Abstract] OR ""depressiveness""[Title/Abstract] OR ""depressives""[Title/Abstract]) AND ""Neurotic""[Title/Abstract]) OR ""neurotic depression""[Title/Abstract] OR ""neurotic depressions""[Title/Abstract] OR ""Melancholia""[Title/Abstract] OR ""Melancholias""[Title/Abstract] OR ""unipolar depression""[Title/Abstract] OR ""depression unipolar""[Title/Abstract] OR ""depressions unipolar""[Title/Abstract] OR ""unipolar depressions""[Title/Abstract])) AND (""Serotonin""[MeSH Terms] OR (""Serotonin""[Title/Abstract] OR ""Hippophaine""[Title/Abstract] OR ((""3""[Title/Abstract] AND ""2-Aminoethyl""[Title/Abstract]) AND ""1h indol 5 ol""[Title/Abstract]) OR ""Enteramine""[Title/Abstract] OR ""5-HT""[Title/Abstract] OR ""5-Hydroxytryptamine""[Title/Abstract] OR ""5-Hydroxytryptamine""[Title/Abstract] OR ""Hydroxytryptamine""[Title/Abstract]) OR (""lipoproteins, hdl""[MeSH Terms] OR (""hdl lipoproteins""[Title/Abstract] OR ""high density lipoprotein""[Title/Abstract] OR ""lipoprotein high density""[Title/Abstract] OR ""high density lipoproteins""[Title/Abstract] OR ""high density lipoproteins""[Title/Abstract] OR ""lipoproteins high density""[Title/Abstract] OR ""alpha-Lipoproteins""[Title/Abstract] OR ""alpha-Lipoproteins""[Title/Abstract] OR ""heavy lipoproteins""[Title/Abstract] OR ((""lipoprotein s""[Title/Abstract] OR ""lipoproteine""[Title/Abstract] OR ""Lipoproteins""[MeSH Terms] OR ""Lipoproteins""[Title/Abstract] OR ""Lipoprotein""[Title/Abstract]) AND ""Heavy""[Title/Abstract]) OR ""high density lipoprotein""[Title/Abstract] OR ""density lipoprotein high""[Title/Abstract] OR ""lipoprotein high density""[Title/Abstract] OR ""alpha-Lipoprotein""[Title/Abstract] OR ""alpha-Lipoprotein""[Title/Abstract] OR ""alpha 1 lipoprotein""[Title/Abstract])) OR (""cholesterol, ldl""[MeSH Terms] OR (""low density lipoprotein cholesterol""[Title/Abstract] OR ""beta lipoprotein cholesterol""[Title/Abstract] OR ""cholesterol beta lipoprotein""[Title/Abstract] OR ""beta lipoprotein cholesterol""[Title/Abstract] OR ""ldl cholesterol""[Title/Abstract] OR ""cholesteryl linoleate ldl""[Title/Abstract] OR ""ldl cholesteryl linoleate""[Title/Abstract])) OR (""cholesterol, vldl""[MeSH Terms] OR (""vldl cholesterol""[Title/Abstract] OR ""pre beta lipoprotein cholesterol""[Title/Abstract] OR ""cholesterol pre beta lipoprotein""[Title/Abstract] OR ""pre beta lipoprotein cholesterol""[Title/Abstract] OR ""very low density lipoprotein cholesterol""[Title/Abstract] OR ""prebetalipoprotein cholesterol""[Title/Abstract] OR ((""Cholesterol""[MeSH Terms] OR ""Cholesterol""[Title/Abstract] OR ""cholesterol s""[Title/Abstract] OR ""cholesterole""[Title/Abstract] OR ""cholesterols""[Title/Abstract]) AND ""Prebetalipoprotein""[Title/Abstract])))) AND (""Longitudinal Studies""[MeSH Terms] OR (""longitudinal""[Title/Abstract] OR ""prospective""[Title/Abstract] OR ""follow-up""[Title/Abstract] OR ""followup""[Title/Abstract]))" 913 8:29:02

28 ("Longitudinal Studies"[Mesh]) OR ((((longitudinal[Title/Abstract]) OR (prospective[Title/Abstract])) OR (follow-up[Title/Abstract])) OR (followup[Title/Abstract])) """Longitudinal Studies""[MeSH Terms] OR ""longitudinal""[Title/Abstract] OR ""prospective""[Title/Abstract] OR ""follow-up""[Title/Abstract] OR ""followup""[Title/Abstract]" "2,186,199" 8:22:45

27 (((longitudinal[Title/Abstract]) OR (prospective[Title/Abstract])) OR (follow-up[Title/Abstract])) OR (followup[Title/Abstract]) """longitudinal""[Title/Abstract] OR ""prospective""[Title/Abstract] OR ""follow-up""[Title/Abstract] OR ""followup""[Title/Abstract]" "2,142,791" 8:22:15

26 """Longitudinal Studies""[Mesh]" Most Recent """Longitudinal Studies""[MeSH Terms]" "171,261" 8:18:45

25 "((((""Serotonin""[Mesh]) OR ((((((((Serotonin[Title/Abstract]) OR (Hippophaine[Title/Abstract])) OR (3-(2-Aminoethyl)-1H-indol-5-ol[Title/Abstract])) OR (Enteramine[Title/Abstract])) OR (5-HT[Title/Abstract])) OR (5-Hydroxytryptamine[Title/Abstract])) OR (5 Hydroxytryptamine[Title/Abstract])) OR (Hydroxytryptamine[Title/Abstract]))) OR ((""Lipoproteins, HDL""[Mesh]) OR ((((((((((((((((HDL Lipoproteins[Title/Abstract]) OR (High-Density Lipoprotein[Title/Abstract])) OR (Lipoprotein, High-Density[Title/Abstract])) OR (High-Density Lipoproteins[Title/Abstract])) OR (High Density Lipoproteins[Title/Abstract])) OR (Lipoproteins, High-Density[Title/Abstract])) OR (alpha-Lipoproteins[Title/Abstract])) OR (alpha-Lipoproteins[Title/Abstract])) OR (Heavy Lipoproteins[Title/Abstract])) OR (Lipoproteins, Heavy[Title/Abstract])) OR (High Density Lipoprotein[Title/Abstract])) OR (Density Lipoprotein, High[Title/Abstract])) OR (Lipoprotein, High Density[Title/Abstract])) OR (alpha-Lipoprotein[Title/Abstract])) OR (alpha Lipoprotein[Title/Abstract])) OR (alpha-1 Lipoprotein[Title/Abstract])))) OR ((""Cholesterol, LDL""[Mesh]) OR (((((((Low Density Lipoprotein Cholesterol[Title/Abstract]) OR (beta-Lipoprotein Cholesterol[Title/Abstract])) OR (Cholesterol, beta-Lipoprotein[Title/Abstract])) OR (beta Lipoprotein Cholesterol[Title/Abstract])) OR (LDL Cholesterol[Title/Abstract])) OR (Cholesteryl Linoleate, LDL[Title/Abstract])) OR (LDL Cholesteryl Linoleate[Title/Abstract])))) OR ((""Cholesterol, VLDL""[Mesh]) OR (((((((VLDL Cholesterol[Title/Abstract]) OR (Pre-beta-Lipoprotein Cholesterol[Title/Abstract])) OR (Cholesterol, Pre-beta-Lipoprotein[Title/Abstract])) OR (Pre beta Lipoprotein Cholesterol[Title/Abstract])) OR (Very Low Density Lipoprotein Cholesterol[Title/Abstract])) OR (Prebetalipoprotein Cholesterol[Title/Abstract])) OR (Cholesterol, Prebetalipoprotein[Title/Abstract])))" """Serotonin""[MeSH Terms] OR (""Serotonin""[Title/Abstract] OR ""Hippophaine""[Title/Abstract] OR ((""3""[Title/Abstract] AND ""2-Aminoethyl""[Title/Abstract]) AND ""1h indol 5 ol""[Title/Abstract]) OR ""Enteramine""[Title/Abstract] OR ""5-HT""[Title/Abstract] OR ""5-Hydroxytryptamine""[Title/Abstract] OR ""5-Hydroxytryptamine""[Title/Abstract] OR ""Hydroxytryptamine""[Title/Abstract]) OR (""lipoproteins, hdl""[MeSH Terms] OR (""hdl lipoproteins""[Title/Abstract] OR ""high density lipoprotein""[Title/Abstract] OR ""lipoprotein high density""[Title/Abstract] OR ""high density lipoproteins""[Title/Abstract] OR ""high density lipoproteins""[Title/Abstract] OR ""lipoproteins high density""[Title/Abstract] OR ""alpha-Lipoproteins""[Title/Abstract] OR ""alpha-Lipoproteins""[Title/Abstract] OR ""heavy lipoproteins""[Title/Abstract] OR ((""lipoprotein s""[Title/Abstract] OR ""lipoproteine""[Title/Abstract] OR ""Lipoproteins""[MeSH Terms] OR ""Lipoproteins""[Title/Abstract] OR ""Lipoprotein""[Title/Abstract]) AND ""Heavy""[Title/Abstract]) OR ""high density lipoprotein""[Title/Abstract] OR ""density lipoprotein high""[Title/Abstract] OR ""lipoprotein high density""[Title/Abstract] OR ""alpha-Lipoprotein""[Title/Abstract] OR ""alpha-Lipoprotein""[Title/Abstract] OR ""alpha 1 lipoprotein""[Title/Abstract])) OR (""cholesterol, ldl""[MeSH Terms] OR (""low density lipoprotein cholesterol""[Title/Abstract] OR ""beta lipoprotein cholesterol""[Title/Abstract] OR ""cholesterol beta lipoprotein""[Title/Abstract] OR ""beta lipoprotein cholesterol""[Title/Abstract] OR ""ldl cholesterol""[Title/Abstract] OR ""cholesteryl linoleate ldl""[Title/Abstract] OR ""ldl cholesteryl linoleate""[Title/Abstract])) OR (""cholesterol, vldl""[MeSH Terms] OR (""vldl cholesterol""[Title/Abstract] OR ""pre beta lipoprotein cholesterol""[Title/Abstract] OR ""cholesterol pre beta lipoprotein""[Title/Abstract] OR ""pre beta lipoprotein cholesterol""[Title/Abstract] OR ""very low density lipoprotein cholesterol""[Title/Abstract] OR ""prebetalipoprotein cholesterol""[Title/Abstract] OR ((""Cholesterol""[MeSH Terms] OR ""Cholesterol""[Title/Abstract] OR ""cholesterol s""[Title/Abstract] OR ""cholesterole""[Title/Abstract] OR ""cholesterols""[Title/Abstract]) AND ""Prebetalipoprotein""[Title/Abstract])))" "276,162" 8:04:31

24 "(""Cholesterol, VLDL""[Mesh]) OR (((((((VLDL Cholesterol[Title/Abstract]) OR (Pre-beta-Lipoprotein Cholesterol[Title/Abstract])) OR (Cholesterol, Pre-beta-Lipoprotein[Title/Abstract])) OR (Pre beta Lipoprotein Cholesterol[Title/Abstract])) OR (Very Low Density Lipoprotein Cholesterol[Title/Abstract])) OR (Prebetalipoprotein Cholesterol[Title/Abstract])) OR (Cholesterol, Prebetalipoprotein[Title/Abstract]))" """cholesterol, vldl""[MeSH Terms] OR (""vldl cholesterol""[Title/Abstract] OR ""pre beta lipoprotein cholesterol""[Title/Abstract] OR ""cholesterol pre beta lipoprotein""[Title/Abstract] OR ""pre beta lipoprotein cholesterol""[Title/Abstract] OR ""very low density lipoprotein cholesterol""[Title/Abstract] OR ""prebetalipoprotein cholesterol""[Title/Abstract] OR ((""Cholesterol""[MeSH Terms] OR ""Cholesterol""[Title/Abstract] OR ""cholesterol s""[Title/Abstract] OR ""cholesterole""[Title/Abstract] OR ""cholesterols""[Title/Abstract]) AND ""Prebetalipoprotein""[Title/Abstract]))" "4,797" 23:40:05

23 "((((((VLDL Cholesterol[Title/Abstract]) OR (Pre-beta-Lipoprotein Cholesterol[Title/Abstract])) OR (Cholesterol, Pre-beta-Lipoprotein[Title/Abstract])) OR (Pre beta Lipoprotein Cholesterol[Title/Abstract])) OR (Very Low Density Lipoprotein Cholesterol[Title/Abstract])) OR (Prebetalipoprotein Cholesterol[Title/Abstract])) OR (Cholesterol, Prebetalipoprotein[Title/Abstract])" """vldl cholesterol""[Title/Abstract] OR ""pre beta lipoprotein cholesterol""[Title/Abstract] OR ""cholesterol pre beta lipoprotein""[Title/Abstract] OR ""pre beta lipoprotein cholesterol""[Title/Abstract] OR ""very low density lipoprotein cholesterol""[Title/Abstract] OR ""prebetalipoprotein cholesterol""[Title/Abstract] OR ((""Cholesterol""[MeSH Terms] OR ""Cholesterol""[Title/Abstract] OR ""cholesterol s""[Title/Abstract] OR ""cholesterole""[Title/Abstract] OR ""cholesterols""[Title/Abstract]) AND ""Prebetalipoprotein""[Title/Abstract])" "3,358" 23:39:47

22 """Cholesterol, VLDL""[Mesh]" Most Recent """cholesterol, vldl""[MeSH Terms]" "2,065" 23:39:06

21 "(""Cholesterol, LDL""[Mesh]) OR (((((((Low Density Lipoprotein Cholesterol[Title/Abstract]) OR (beta-Lipoprotein Cholesterol[Title/Abstract])) OR (Cholesterol, beta-Lipoprotein[Title/Abstract])) OR (beta Lipoprotein Cholesterol[Title/Abstract])) OR (LDL Cholesterol[Title/Abstract])) OR (Cholesteryl Linoleate, LDL[Title/Abstract])) OR (LDL Cholesteryl Linoleate[Title/Abstract]))" """cholesterol, ldl""[MeSH Terms] OR ""low density lipoprotein cholesterol""[Title/Abstract] OR ""beta lipoprotein cholesterol""[Title/Abstract] OR ""cholesterol beta lipoprotein""[Title/Abstract] OR ""beta lipoprotein cholesterol""[Title/Abstract] OR ""ldl cholesterol""[Title/Abstract] OR ""cholesteryl linoleate ldl""[Title/Abstract] OR ""ldl cholesteryl linoleate""[Title/Abstract]" "71,048" 23:38:12

20 "((((((Low Density Lipoprotein Cholesterol[Title/Abstract]) OR (beta-Lipoprotein Cholesterol[Title/Abstract])) OR (Cholesterol, beta-Lipoprotein[Title/Abstract])) OR (beta Lipoprotein Cholesterol[Title/Abstract])) OR (LDL Cholesterol[Title/Abstract])) OR (Cholesteryl Linoleate, LDL[Title/Abstract])) OR (LDL Cholesteryl Linoleate[Title/Abstract])" """low density lipoprotein cholesterol""[Title/Abstract] OR ""beta lipoprotein cholesterol""[Title/Abstract] OR ""cholesterol beta lipoprotein""[Title/Abstract] OR ""beta lipoprotein cholesterol""[Title/Abstract] OR ""ldl cholesterol""[Title/Abstract] OR ""cholesteryl linoleate ldl""[Title/Abstract] OR ""ldl cholesteryl linoleate""[Title/Abstract]" "58,399" 23:37:41

19 """Cholesterol, LDL""[Mesh]" Most Recent """cholesterol, ldl""[MeSH Terms]" "32,900" 23:36:46

18 ("Triglycerides"[Mesh]) OR (((Triacylglycerols[Title/Abstract]) OR (Triacylglycerol[Title/Abstract])) OR (Triglyceride[Title/Abstract])) """Triglycerides""[MeSH Terms] OR ""Triacylglycerols""[Title/Abstract] OR ""Triacylglycerol""[Title/Abstract] OR ""Triglyceride""[Title/Abstract]" "141,142" 23:35:39

17 ((Triacylglycerols[Title/Abstract]) OR (Triacylglycerol[Title/Abstract])) OR (Triglyceride[Title/Abstract]) """Triacylglycerols""[Title/Abstract] OR ""Triacylglycerol""[Title/Abstract] OR ""Triglyceride""[Title/Abstract]" "93,300" 23:35:05

16 """Triglycerides""[Mesh]" Most Recent """Triglycerides""[MeSH Terms]" "86,228" 23:34:26

15 "(""Lipoproteins, HDL""[Mesh]) OR ((((((((((((((((HDL Lipoproteins[Title/Abstract]) OR (High-Density Lipoprotein[Title/Abstract])) OR (Lipoprotein, High-Density[Title/Abstract])) OR (High-Density Lipoproteins[Title/Abstract])) OR (High Density Lipoproteins[Title/Abstract])) OR (Lipoproteins, High-Density[Title/Abstract])) OR (alpha-Lipoproteins[Title/Abstract])) OR (alpha-Lipoproteins[Title/Abstract])) OR (Heavy Lipoproteins[Title/Abstract])) OR (Lipoproteins, Heavy[Title/Abstract])) OR (High Density Lipoprotein[Title/Abstract])) OR (Density Lipoprotein, High[Title/Abstract])) OR (Lipoprotein, High Density[Title/Abstract])) OR (alpha-Lipoprotein[Title/Abstract])) OR (alpha Lipoprotein[Title/Abstract])) OR (alpha-1 Lipoprotein[Title/Abstract]))" """lipoproteins, hdl""[MeSH Terms] OR (""hdl lipoproteins""[Title/Abstract] OR ""high density lipoprotein""[Title/Abstract] OR ""lipoprotein high density""[Title/Abstract] OR ""high density lipoproteins""[Title/Abstract] OR ""high density lipoproteins""[Title/Abstract] OR ""lipoproteins high density""[Title/Abstract] OR ""alpha-Lipoproteins""[Title/Abstract] OR ""alpha-Lipoproteins""[Title/Abstract] OR ""heavy lipoproteins""[Title/Abstract] OR ((""lipoprotein s""[Title/Abstract] OR ""lipoproteine""[Title/Abstract] OR ""Lipoproteins""[MeSH Terms] OR ""Lipoproteins""[Title/Abstract] OR ""Lipoprotein""[Title/Abstract]) AND ""Heavy""[Title/Abstract]) OR ""high density lipoprotein""[Title/Abstract] OR ""density lipoprotein high""[Title/Abstract] OR ""lipoprotein high density""[Title/Abstract] OR ""alpha-Lipoprotein""[Title/Abstract] OR ""alpha-Lipoprotein""[Title/Abstract] OR ""alpha 1 lipoprotein""[Title/Abstract])" "90,365" 23:33:06

14 "(((((((((((((((HDL Lipoproteins[Title/Abstract]) OR (High-Density Lipoprotein[Title/Abstract])) OR (Lipoprotein, High-Density[Title/Abstract])) OR (High-Density Lipoproteins[Title/Abstract])) OR (High Density Lipoproteins[Title/Abstract])) OR (Lipoproteins, High-Density[Title/Abstract])) OR (alpha-Lipoproteins[Title/Abstract])) OR (alpha-Lipoproteins[Title/Abstract])) OR (Heavy Lipoproteins[Title/Abstract])) OR (Lipoproteins, Heavy[Title/Abstract])) OR (High Density Lipoprotein[Title/Abstract])) OR (Density Lipoprotein, High[Title/Abstract])) OR (Lipoprotein, High Density[Title/Abstract])) OR (alpha-Lipoprotein[Title/Abstract])) OR (alpha Lipoprotein[Title/Abstract])) OR (alpha-1 Lipoprotein[Title/Abstract])" """hdl lipoproteins""[Title/Abstract] OR ""high density lipoprotein""[Title/Abstract] OR ""lipoprotein high density""[Title/Abstract] OR ""high density lipoproteins""[Title/Abstract] OR ""high density lipoproteins""[Title/Abstract] OR ""lipoproteins high density""[Title/Abstract] OR ""alpha-Lipoproteins""[Title/Abstract] OR ""alpha-Lipoproteins""[Title/Abstract] OR ""heavy lipoproteins""[Title/Abstract] OR ((""lipoprotein s""[Title/Abstract] OR ""lipoproteine""[Title/Abstract] OR ""Lipoproteins""[MeSH Terms] OR ""Lipoproteins""[Title/Abstract] OR ""Lipoprotein""[Title/Abstract]) AND ""Heavy""[Title/Abstract]) OR ""high density lipoprotein""[Title/Abstract] OR ""density lipoprotein high""[Title/Abstract] OR ""lipoprotein high density""[Title/Abstract] OR ""alpha-Lipoprotein""[Title/Abstract] OR ""alpha-Lipoprotein""[Title/Abstract] OR ""alpha 1 lipoprotein""[Title/Abstract]" "68,345" 23:32:37

13 """Lipoproteins, HDL""[Mesh]" Most Recent """lipoproteins, hdl""[MeSH Terms]" "48,646" 23:30:51

12 ("Cholesterol"[Mesh]) OR (Epicholesterol[Title/Abstract]) """Cholesterol""[MeSH Terms] OR ""Epicholesterol""[Title/Abstract]" "176,958" 23:30:18

11 Epicholesterol[Title/Abstract] """Epicholesterol""[Title/Abstract]" 88 23:19:37

10 """Cholesterol""[Mesh]" Most Recent """Cholesterol""[MeSH Terms]" "176,940" 23:19:11

9 ("Serotonin"[Mesh]) OR ((((((((Serotonin[Title/Abstract]) OR (Hippophaine[Title/Abstract])) OR (3-(2-Aminoethyl)-1H-indol-5-ol[Title/Abstract])) OR (Enteramine[Title/Abstract])) OR (5-HT[Title/Abstract])) OR (5-Hydroxytryptamine[Title/Abstract])) OR (5 Hydroxytryptamine[Title/Abstract])) OR (Hydroxytryptamine[Title/Abstract])) """Serotonin""[MeSH Terms] OR (""Serotonin""[Title/Abstract] OR ""Hippophaine""[Title/Abstract] OR ((""3""[Title/Abstract] AND ""2-Aminoethyl""[Title/Abstract]) AND ""1h indol 5 ol""[Title/Abstract]) OR ""Enteramine""[Title/Abstract] OR ""5-HT""[Title/Abstract] OR ""5-Hydroxytryptamine""[Title/Abstract] OR ""5-Hydroxytryptamine""[Title/Abstract] OR ""Hydroxytryptamine""[Title/Abstract])" "146,495" 23:18:26

7 (((((((Serotonin[Title/Abstract]) OR (Hippophaine[Title/Abstract])) OR (3-(2-Aminoethyl)-1H-indol-5-ol[Title/Abstract])) OR (Enteramine[Title/Abstract])) OR (5-HT[Title/Abstract])) OR (5-Hydroxytryptamine[Title/Abstract])) OR (5 Hydroxytryptamine[Title/Abstract])) OR (Hydroxytryptamine[Title/Abstract]) """Serotonin""[Title/Abstract] OR ""Hippophaine""[Title/Abstract] OR ((""3""[Title/Abstract] AND ""2-Aminoethyl""[Title/Abstract]) AND ""1h indol 5 ol""[Title/Abstract]) OR ""Enteramine""[Title/Abstract] OR ""5-HT""[Title/Abstract] OR ""5-Hydroxytryptamine""[Title/Abstract] OR ""5-Hydroxytryptamine""[Title/Abstract] OR ""Hydroxytryptamine""[Title/Abstract]" "129,791" 23:16:30

6 """Serotonin""[Mesh]" Most Recent """Serotonin""[MeSH Terms]" "71,939" 23:15:14

5 "(""Depressive Disorder""[Mesh]) OR ((((((((((((((((((((((((((Depressive Disorder[Title/Abstract]) OR (Depressive Disorders[Title/Abstract])) OR (Disorder, Depressive[Title/Abstract])) OR (Disorders, Depressive[Title/Abstract])) OR (Neurosis, Depressive[Title/Abstract])) OR (Depressive Neuroses[Title/Abstract])) OR (Depressive Neurosis[Title/Abstract])) OR (Neuroses, Depressive[Title/Abstract])) OR (Depression, Endogenous[Title/Abstract])) OR (Depressions, Endogenous[Title/Abstract])) OR (Endogenous Depression[Title/Abstract])) OR (Endogenous Depressions[Title/Abstract])) OR (Depressive Syndrome[Title/Abstract])) OR (Depressive Syndromes[Title/Abstract])) OR (Syndrome, Depressive[Title/Abstract])) OR (Syndromes, Depressive[Title/Abstract])) OR (Depression, Neurotic[Title/Abstract])) OR (Depressions, Neurotic[Title/Abstract])) OR (Neurotic Depression[Title/Abstract])) OR (Neurotic Depressions[Title/Abstract])) OR (Melancholia[Title/Abstract])) OR (Melancholias[Title/Abstract])) OR (Unipolar Depression[Title/Abstract])) OR (Depression, Unipolar[Title/Abstract])) OR (Depressions, Unipolar[Title/Abstract])) OR (Unipolar Depressions[Title/Abstract]))" """Depressive Disorder""[MeSH Terms] OR (""Depressive Disorder""[Title/Abstract] OR ""depressive disorders""[Title/Abstract] OR ""disorder depressive""[Title/Abstract] OR ""disorders depressive""[Title/Abstract] OR ""neurosis depressive""[Title/Abstract] OR ""depressive neuroses""[Title/Abstract] OR ""depressive neurosis""[Title/Abstract] OR ""neuroses depressive""[Title/Abstract] OR ""depression endogenous""[Title/Abstract] OR ""depressions endogenous""[Title/Abstract] OR ""endogenous depression""[Title/Abstract] OR ""endogenous depressions""[Title/Abstract] OR ""depressive syndrome""[Title/Abstract] OR ""depressive syndromes""[Title/Abstract] OR ""syndrome depressive""[Title/Abstract] OR ""syndromes depressive""[Title/Abstract] OR ""depression neurotic""[Title/Abstract] OR ((""depressed""[Title/Abstract] OR ""Depression""[MeSH Terms] OR ""Depression""[Title/Abstract] OR ""Depressions""[Title/Abstract] OR ""depression s""[Title/Abstract] OR ""Depressive Disorder""[MeSH Terms] OR (""Depressive""[Title/Abstract] AND ""Disorder""[Title/Abstract]) OR ""Depressive Disorder""[Title/Abstract] OR ""depressivity""[Title/Abstract] OR ""Depressive""[Title/Abstract] OR ""depressively""[Title/Abstract] OR ""depressiveness""[Title/Abstract] OR ""depressives""[Title/Abstract]) AND ""Neurotic""[Title/Abstract]) OR ""neurotic depression""[Title/Abstract] OR ""neurotic depressions""[Title/Abstract] OR ""Melancholia""[Title/Abstract] OR ""Melancholias""[Title/Abstract] OR ""unipolar depression""[Title/Abstract] OR ""depression unipolar""[Title/Abstract] OR ""depressions unipolar""[Title/Abstract] OR ""unipolar depressions""[Title/Abstract])" "147,994" 23:14:04

4 "(((((((((((((((((((((((((Depressive Disorder[Title/Abstract]) OR (Depressive Disorders[Title/Abstract])) OR (Disorder, Depressive[Title/Abstract])) OR (Disorders, Depressive[Title/Abstract])) OR (Neurosis, Depressive[Title/Abstract])) OR (Depressive Neuroses[Title/Abstract])) OR (Depressive Neurosis[Title/Abstract])) OR (Neuroses, Depressive[Title/Abstract])) OR (Depression, Endogenous[Title/Abstract])) OR (Depressions, Endogenous[Title/Abstract])) OR (Endogenous Depression[Title/Abstract])) OR (Endogenous Depressions[Title/Abstract])) OR (Depressive Syndrome[Title/Abstract])) OR (Depressive Syndromes[Title/Abstract])) OR (Syndrome, Depressive[Title/Abstract])) OR (Syndromes, Depressive[Title/Abstract])) OR (Depression, Neurotic[Title/Abstract])) OR (Depressions, Neurotic[Title/Abstract])) OR (Neurotic Depression[Title/Abstract])) OR (Neurotic Depressions[Title/Abstract])) OR (Melancholia[Title/Abstract])) OR (Melancholias[Title/Abstract])) OR (Unipolar Depression[Title/Abstract])) OR (Depression, Unipolar[Title/Abstract])) OR (Depressions, Unipolar[Title/Abstract])) OR (Unipolar Depressions[Title/Abstract])" """depressive disorder""[Title/Abstract] OR ""depressive disorders""[Title/Abstract] OR ""disorder depressive""[Title/Abstract] OR ""disorders depressive""[Title/Abstract] OR ""neurosis depressive""[Title/Abstract] OR ""depressive neuroses""[Title/Abstract] OR ""depressive neurosis""[Title/Abstract] OR ""neuroses depressive""[Title/Abstract] OR ""depression endogenous""[Title/Abstract] OR ""depressions endogenous""[Title/Abstract] OR ""endogenous depression""[Title/Abstract] OR ""endogenous depressions""[Title/Abstract] OR ""depressive syndrome""[Title/Abstract] OR ""depressive syndromes""[Title/Abstract] OR ""syndrome depressive""[Title/Abstract] OR ""syndromes depressive""[Title/Abstract] OR ""depression neurotic""[Title/Abstract] OR ((""depressed""[Title/Abstract] OR ""Depression""[MeSH Terms] OR ""Depression""[Title/Abstract] OR ""Depressions""[Title/Abstract] OR ""depression s""[Title/Abstract] OR ""depressive disorder""[MeSH Terms] OR (""Depressive""[Title/Abstract] AND ""Disorder""[Title/Abstract]) OR ""depressive disorder""[Title/Abstract] OR ""depressivity""[Title/Abstract] OR ""Depressive""[Title/Abstract] OR ""depressively""[Title/Abstract] OR ""depressiveness""[Title/Abstract] OR ""depressives""[Title/Abstract]) AND ""Neurotic""[Title/Abstract]) OR ""neurotic depression""[Title/Abstract] OR ""neurotic depressions""[Title/Abstract] OR ""Melancholia""[Title/Abstract] OR ""Melancholias""[Title/Abstract] OR ""unipolar depression""[Title/Abstract] OR ""depression unipolar""[Title/Abstract] OR ""depressions unipolar""[Title/Abstract] OR ""unipolar depressions""[Title/Abstract]" "59,985" 23:12:54

3 """Depressive Disorder""[Mesh]" Most Recent """Depressive Disorder""[MeSH Terms]" "124,597" 23:08:46

**WOS Resarch : 1351**

权限 # 检索式 数据库 检索结果 运行日期

"

- WOS.IC: 1993 to 2024

- WOS.CCR: 1985 to 2024

- WOS.SCI: 1975 to 2024

- WOS.AHCI: 1975 to 2024

- WOS.BHCI: 2005 to 2024

- WOS.BSCI: 2005 to 2024

- WOS.ESCI: 2019 to 2024

- WOS.ISTP: 1990 to 2024

- WOS.SSCI: 1965 to 2024

- WOS.ISSHP: 1990 to 2024" 1 "TS=(Depressive Disorder Depressive Disorder OR Depressive Disorders OR Disorder, Depressive OR Disorders, Depressive OR Neurosis, Depressive OR Depressive Neuroses OR Depressive Neurosis OR Neuroses, Depressive OR Depression, Endogenous OR Depressions, Endogenous OR Endogenous Depression OR Endogenous Depressions OR Depressive Syndrome OR Depressive Syndromes OR Syndrome, Depressive OR Syndromes, Depressive OR Depression, Neurotic OR Depressions, Neurotic OR Neurotic Depression OR Neurotic Depressions OR Melancholia OR Melancholias OR Unipolar Depression OR Depression, Unipolar OR Depressions, Unipolar OR Unipolar Depressions

) " Web of Science 核心合集 132424 Sat May 04 2024 20:36:10 GMT+0800 (中国标准时间)

"

- WOS.IC: 1993 to 2024

- WOS.CCR: 1985 to 2024

- WOS.SCI: 1975 to 2024

- WOS.AHCI: 1975 to 2024

- WOS.BHCI: 2005 to 2024

- WOS.BSCI: 2005 to 2024

- WOS.ESCI: 2019 to 2024

- WOS.ISTP: 1990 to 2024

- WOS.SSCI: 1965 to 2024

- WOS.ISSHP: 1990 to 2024" 2 "TS=(Serotonin OR Serotonin OR Hippophaine OR 3 (2 Aminoethyl) 1H indol 5 ol OR Enteramine OR 5 HT OR 5 Hydroxytryptamine OR 5 Hydroxytryptamine OR Hydroxytryptamine OR Epicholesterol

) " Web of Science 核心合集 162625 Sat May 04 2024 20:37:14 GMT+0800 (中国标准时间)

"

- WOS.IC: 1993 to 2024

- WOS.CCR: 1985 to 2024

- WOS.SCI: 1975 to 2024

- WOS.AHCI: 1975 to 2024

- WOS.BHCI: 2005 to 2024

- WOS.BSCI: 2005 to 2024

- WOS.ESCI: 2019 to 2024

- WOS.ISTP: 1990 to 2024

- WOS.SSCI: 1965 to 2024

- WOS.ISSHP: 1990 to 2024" 3 "TS=(cholesterol OR Epicholesterol

) " Web of Science 核心合集 330006 Sat May 04 2024 20:37:53 GMT+0800 (中国标准时间)

"

- WOS.IC: 1993 to 2024

- WOS.CCR: 1985 to 2024

- WOS.SCI: 1975 to 2024

- WOS.AHCI: 1975 to 2024

- WOS.BHCI: 2005 to 2024

- WOS.BSCI: 2005 to 2024

- WOS.ESCI: 2019 to 2024

- WOS.ISTP: 1990 to 2024

- WOS.SSCI: 1965 to 2024

- WOS.ISSHP: 1990 to 2024" 4 "TS=(Cholesterol, HDL OR HDL Lipoproteins OR High-Density Lipoprotein OR Lipoprotein, High-Density OR High-Density Lipoproteins OR High Density Lipoproteins OR Lipoproteins, High-Density OR alpha-Lipoproteins OR alpha Lipoproteins OR Heavy Lipoproteins OR Lipoproteins, Heavy OR High Density Lipoprotein OR Density Lipoprotein, High OR Lipoprotein, High Density OR alpha-Lipoprotein OR alpha Lipoprotein OR alpha 1 Lipoprotein

) " Web of Science 核心合集 146653 Sat May 04 2024 20:38:27 GMT+0800 (中国标准时间)

"

- WOS.IC: 1993 to 2024

- WOS.CCR: 1985 to 2024

- WOS.SCI: 1975 to 2024

- WOS.AHCI: 1975 to 2024

- WOS.BHCI: 2005 to 2024

- WOS.BSCI: 2005 to 2024

- WOS.ESCI: 2019 to 2024

- WOS.ISTP: 1990 to 2024

- WOS.SSCI: 1965 to 2024

- WOS.ISSHP: 1990 to 2024" 5 "TS=(triglyceride OR Triacylglycerols OR Triacylglycerol OR Triglyceride

) " Web of Science 核心合集 166956 Sat May 04 2024 20:39:07 GMT+0800 (中国标准时间)

"

- WOS.IC: 1993 to 2024

- WOS.CCR: 1985 to 2024

- WOS.SCI: 1975 to 2024

- WOS.AHCI: 1975 to 2024

- WOS.BHCI: 2005 to 2024

- WOS.BSCI: 2005 to 2024

- WOS.ESCI: 2019 to 2024

- WOS.ISTP: 1990 to 2024

- WOS.SSCI: 1965 to 2024

- WOS.ISSHP: 1990 to 2024" 6 "TS=(Low density lipoprotein cholesterol OR Low Density Lipoprotein Cholesterol OR beta-Lipoprotein Cholesterol OR Cholesterol, beta-Lipoprotein OR beta Lipoprotein Cholesterol OR LDL Cholesterol OR Cholesteryl Linoleate, LDL OR LDL Cholesteryl Linoleate

) " Web of Science 核心合集 111556 Sat May 04 2024 20:39:38 GMT+0800 (中国标准时间)

"

- WOS.IC: 1993 to 2024

- WOS.CCR: 1985 to 2024

- WOS.SCI: 1975 to 2024

- WOS.AHCI: 1975 to 2024

- WOS.BHCI: 2005 to 2024

- WOS.BSCI: 2005 to 2024

- WOS.ESCI: 2019 to 2024

- WOS.ISTP: 1990 to 2024

- WOS.SSCI: 1965 to 2024

- WOS.ISSHP: 1990 to 2024" 7 "TS=(Very Low density lipoprotein cholesterol OR VLDL Cholesterol OR Pre-beta-Lipoprotein Cholesterol OR Cholesterol, Pre-beta-Lipoprotein OR Pre beta Lipoprotein Cholesterol OR Very Low Density Lipoprotein Cholesterol OR Prebetalipoprotein Cholesterol OR Cholesterol, Prebetalipoprotein

) " Web of Science 核心合集 14589 Sat May 04 2024 20:40:19 GMT+0800 (中国标准时间)

"

- WOS.IC: 1993 to 2024

- WOS.CCR: 1985 to 2024

- WOS.SCI: 1975 to 2024

- WOS.AHCI: 1975 to 2024

- WOS.BHCI: 2005 to 2024

- WOS.BSCI: 2005 to 2024

- WOS.ESCI: 2019 to 2024

- WOS.ISTP: 1990 to 2024

- WOS.SSCI: 1965 to 2024

- WOS.ISSHP: 1990 to 2024" 8 "#2 OR #3 OR #4 OR #5 OR #6 OR #7 " Web of Science 核心合集 603849 Sat May 04 2024 20:41:50 GMT+0800 (中国标准时间)

"

- WOS.IC: 1993 to 2024

- WOS.CCR: 1985 to 2024

- WOS.SCI: 1975 to 2024

- WOS.AHCI: 1975 to 2024

- WOS.BHCI: 2005 to 2024

- WOS.BSCI: 2005 to 2024

- WOS.ESCI: 2019 to 2024

- WOS.ISTP: 1990 to 2024

- WOS.SSCI: 1965 to 2024

- WOS.ISSHP: 1990 to 2024" 9 "TS=(longitudinal OR prospective OR follow-up OR followup

) " Web of Science 核心合集 2508230 Sat May 04 2024 20:46:14 GMT+0800 (中国标准时间)

"

- WOS.IC: 1993 to 2024

- WOS.CCR: 1985 to 2024

- WOS.SCI: 1975 to 2024

- WOS.AHCI: 1975 to 2024

- WOS.BHCI: 2005 to 2024

- WOS.BSCI: 2005 to 2024

- WOS.ESCI: 2019 to 2024

- WOS.ISTP: 1990 to 2024

- WOS.SSCI: 1965 to 2024

- WOS.ISSHP: 1990 to 2024" 10 "#1 AND #8 AND #9 " Web of Science 核心合集 1351 Sat May 04 2024 20:47:18 GMT+0800 (中国标准时间)
